# Are Biobased Microfibers Less Harmful than Conventional Plastic Microfibers: Evidence from Earthworms

**DOI:** 10.1021/acs.est.4c05856

**Published:** 2024-11-05

**Authors:** W. Courtene-Jones, F. De Falco, F. Burgevin, R. D. Handy, R. C. Thompson

**Affiliations:** † School of Biological and Marine Sciences, University of Plymouth, Drake Circus, Plymouth, Devon PL4 8AA, U.K.; ‡ School of Ocean Science, Bangor University, Anglesey LL59 5AB, U.K.; § School of Geography, Earth and Environmental Sciences, University of Plymouth, Drake Circus, Plymouth, Devon PL4 8AA, U.K.; ∥ Institute for Sustainability, Department of Chemistry, University of Bath, Bath BA2 7AY, U.K.

**Keywords:** Microplastic, Biopolymer, Synthetic fibers, Environmental risk assessment, Hazard assessment, Fecundity

## Abstract

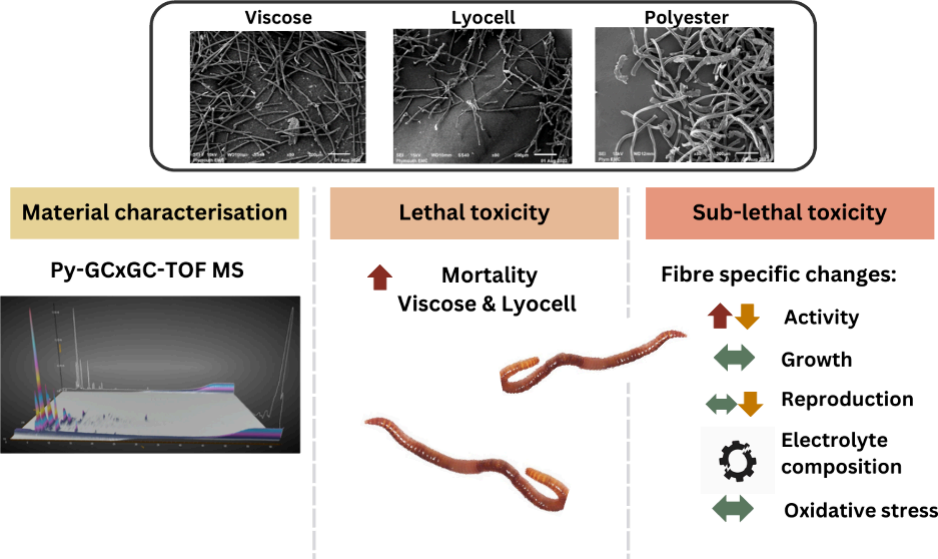

Biobased plastics are sometimes promoted as “environmentally friendly” compared to their conventional petrochemical-based counterparts, but their ecotoxicity is only partially understood. Biobased fibers are widely used in clothing and wet wipes and can accumulate in soils through the application of biosolid fertilizers. This study examined the lethal thresholds and sublethal toxicity of chemically characterized, additive-free, biobased (viscose and lyocell) compared to petrochemical-based (polyester) fibers on the key ecosystem engineer, *Esenia fetida*. Viscose and lyocell had LC_20_ values of 14.00 and 22.66 mg·L^–1^, respectively, and no observed effect concentrations (NOEC) of 0–2.8 mg·L^–1^ (72 h, OECD TG207 filter paper method), while for polyester these were LC_20_ 15.6–31.3 mg·L^–1^ and NOEC 0–15.6 mg·L^–1^. Following 28 days of exposure to soils (OECD TG222) contaminated with environmentally relevant concentrations (100 mg kg^–1^), viscose significantly reduced the mass of progeny compared to polyester. Earthworms exposed to lyocell had a marginal growth reduction (−18%; compared to −11% to −13% in other treatments) linked to increased bioturbation activity. The biobased fibers examined here have greater acute toxicity at high concentrations and broadly similar sublethal effects on *E. fetida* compared to polyester. Our study highlights the importance of detailed testing before advocating specific materials as plastic alternatives/substitutes to conventional plastics.

## Abstract 2

Plastic fibers are known to have detrimental impacts on organisms; here, we show that biobased fibers have the potential to induce greater ecotoxicological effects than traditional plastics with implications for population dynamics.

## 1 Introduction

Over the last few decades, partly in response to the rising concern about (micro)plastic pollution, there has been an increasing motivation to substitute petrochemical-based plastics with biobased and biodegradable alternatives.^1,2^ This has been observed across a range of sectors including the textile industry where 329,000 tonnes of biobased and biodegradable fibers were produced in 2022, occupying the third largest market share after flexible and rigid packaging (696,000 and 376,000 tonnes respectively).^3^

Biobased plastics are entirely, or partly, derived from biomass (including plant, animal, and marine or forestry materials) but are not necessarily biodegradable.^4,5^ Numerous industrial processes, chemical additives (e.g., dyes), and finishing products are used during fiber production, modifying the bioderived material and potentially making the polymers more durable.^6,7^ Indeed, biobased fibers, such as viscose, rayon, and cotton, have been reported in aquatic and terrestrial environments, and in some cases exceed the concentrations of conventional microplastic fibers.^8−10^ Biobased fibers are used in clothing and the manufacture of wet wipes and sanitary products. Fibers shed during the laundering of clothes and the inappropriate flushing of wet wipes and sanitary products enter wastewater treatment facilities where the majority are retained within biosolids in the sewage sludge. Biosolids are applied to agricultural land as a fertilizer which can introduce microplastics, of which >90% are fibers, into soils.^11,12^ It is estimated that annually up to 63,000–430,000 tonnes of microplastic may enter European farmlands,^13^ largely through the application of biosolids. The quantity of biosolids applied to agricultural soils varies between countries; in the UK, 96% of biosolids are used as fertilizers, higher than the European Union average of 50%.^14,15^ Data shows that in England, 16 × 10^9^ MP.t̅^1^ of dry weight sludge are added to agricultural soils annually, which when extrapolated by the average quantity of microplastics contained within them equates to an emission of 1.29 × 10^15^ microplastics per annum.^11^ Consequently, there is the potential for localized introduction and accumulation of microfibers (biobased such as viscose, or petrochemical-based such as polyester) within agricultural soils.

Within soils, earthworms are ecosystem engineers contributing toward nutrient cycling and the creation, oxygenation and irrigation of soil,^16,17^ which modulate key services such as food production. Conventional petrochemical-based fibers have been shown to cause a range of impairments on the survival, growth, reproduction and cellular/biochemical regulation of terrestrial invertebrates, including earthworms.^18−21^ By comparison, there is limited quantification of the biological effects of biobased plastics on earthworms,^22−24^ and a dearth of studies specifically focusing on fibers. Consequently, there are insufficient data on the acute and chronic effects of biobased fibers required for environmental risk assessment.

Globally, fiber markets are expected to increase their production over the next decade. Conventional petrochemical-based fibers dominate the market share, in particular polyester which, in 2022, accounted for 56% (63 million tonnes) of the global fiber produced.^25^ The production of biobased fibers are also projected to increase;^3^ therefore, it is necessary to determine their associated ecological impacts in order to inform risk evaluation. Lyocell and viscose are both manmade cellulose fibers derived from wood pulp but are produced by differing industrial production processes.^26^ In 2022, viscose accounted for ∼80% (5.8 million tonnes) and lyocell 4% (0.3 million tonnes) of all manmade cellulosic fibers.^25^ These three materials were, therefore, selected for testing due to their differing production processes and contribution to overall market shares.

This study aimed to compare the ecotoxicity of undyed biobased (viscose and lyocell) and conventional (polyester) microfibers on the key soil ecosystem engineer, *Eisenia fetida*. Chemical characterization of the fibers generated from textiles, which were supplied directly from the manufacturer, did not detect the presence of any chemical additives or finishing products in the materials. In the absence of acute toxicity data, experiments were initially conducted to determine the lethal threshold of each material, and then a second series of experiments using environmentally relevant concentrations^11,27−29^ examined sublethal effects across a suite of end points including growth and reproductive success. To be clear, this study did not seek to determine the mechanism of toxicity but to examine the individual and likely population level effects of viscose, lyocell, and polyester fibers on *E. fetida* earthworms. The approach followed the Organisation for Economic Cooperation and Development (OECD) standardized test methods as closely as possible to give the data utility in Environmental Risk Assessment.

## 2 Materials and Method

### 2.1 Fiber Generation and Characterization

Two biobased textiles (viscose and lyocell) and one conventional plastic textile (polyester) were considered, each in their undyed forms to eliminate dye from being a potential extraneous variable in ecotoxicity. Viscose and lyocell are both artificially regenerated cellulose fibers, but their production processes are different. According to the manufacturer, both fibers are derived from pulp from renewable wood.^26^ For viscose fibers, the cellulose is derivatized through xanthation with CS2 and then dissolved in a solution of sodium hydroxide. For lyocell fibers, cellulose is directly dissolved in the solvent *N*-methyl morpholine-N-oxide (NMMO, C_5_H_11_NO_2_), which also chemically alters cellulose due to its strong oxidant nature.^30^ Due to environmental problems related to the xanthation, lyocell is often presented as the environmentally friendly alternative to viscose.^30^

Knitted textiles were supplied in 5 × 1 m pieces by Lenzing AG, Austria. To produce fibers of the desired size range, 0.5 cm^2^ pieces were cut from the fabric and were manually cryo-ground under liquid nitrogen using a pestle and mortar. Fibers were size fractionated using stacked stainless-steel sieves, rinsed with 70% ethanol followed by ultrapure Milli-Q water (18.2 Ω), and then suspended in Milli-Q deionized water in separate glass jars. Fibers between 125–300 μm in length were used as this is within the ingestible size range for *Eisenia fetida.*(31) Characterization of the textile material and microfibers are provided in Table 1 and the Supporting Information (Scanning electron microscopy (Figures S1–S3), Fourier transform infrared spectroscopy (Figure S4, Table S1), pyrolysis-gas chromatography × gas chromatography-time-of-flight mass spectrometry (Py-GCxGC-TOF MS) (Figure S5, Tables S2–S4), and powder X-ray diffraction (Figure S6).

**Table 1:** Summary of the Fiber Characteristics

Material	Material description	Material classification	Fiber length (μm)	Material density (g/m^2^)^a^	Material water retention (g/g)^b^
Viscose	Regenerated cellulosic fibers produced from wood pulp	Biobased	125–300	108	0.89
Lyocell	Regenerated cellulosic fibers produced from wood pulp	Biobased	125–300	118	∼0.80–0.94
Polyester	Synthetic fiber, poly(ethylene terephthalate) (PET)	Petrochemical-based	125–300	108	0.12

a Information from manufacturer.

b Data from Okubayashi, Griesser, and Bechtold.^32^

#### 2.1.1 Fourier Transform Infrared Spectroscopy

The composition of the materials (*n* = 3) were investigated with a Fourier transform infrared spectrometer [FTIR; Vertex 70 (Bruker, Germany)], with an attenuated total reflectance accessory (ATR). Spectra were acquired using 32 scans and a resolution of 4 cm^–1^ over the range 4000–400 cm^–1^. The obtained spectra were compared to multiple spectral databases (i.e., Bruker Optics ATR-Polymer Library, KIMW ATR-IR Polymer Library, ATR-FTIR Library Polymer, etc. Supporting Information Section S2).

#### 2.1.2 Py-GCxGC-TOF MS

Pyrolysis coupled with gas chromatography and mass spectrometry represents a powerful tool for analyzing the composition of polymeric materials, due to the identification of the thermal degradation products released during the pyrolysis process.^33,34^ The chemical compositions of textiles (80–100 μg, *n* = 3) were examined using pyrolysis coupled to 2D gas chromatography and time-of-flight mass spectrometry (Py-GCxGC-TOF MS). The instrumentation consisted of a CDS6200 pyroprobe equipped with drop-in-sample chamber (DISC) (CDS Analytical, USA) coupled to an 8890 gas chromatograph (Agilent Technologies, USA) and bench TOF2-TI time-of-flight mass spectrometer (SepSolve Analytical, UK). The GC was equipped with an INSIGHT flow modulator (SepSolve Analytical, UK) for 2D chromatography and with the following columns: a deactivated silica guard column (5 m × 0.25 mm, Restek, UK), a BPX5 fused silica capillary 1D column (stationary phase 5% phenyl–95% dimethyl polysiloxane, 20 m × 0.18 mm i.d., 0.18 μm, Trajan Scientific and Medical, Australia), a BPX50 fused silica capillary 2D column (stationary phase 50% phenyl polysilphenylene-siloxane, 5 m × 0.25 mm i.d., 0.10 μm, Trajan Scientific and Medical, Australia). Each type of textile was analyzed in triplicate, with 80–100 μg of each sample loaded into a quartz tube packed by quartz wool on the open end, and inserted in the DISC. Here the sample was pyrolyzed in single shot at 600 °C for 30 s in He atmosphere. The GC injector was kept at 300 °C with a 150:1 split ratio. The pyrolysis products were eluted in constant flow mode at 0.5 mL min^–1^ (1D column) and 20 mL min^–1^ (2D column) with He as the carrier gas (purity 99.995%) and a modulation period of 4 s. The chromatographic program was: 40 °C isotherm for 6 min, 5 °C min^–1^ up to 300 °C, 300 °C isotherm for 10 min. The time-of-flight spectrometer had an acquisition rate of 50.0 Hz in electron ionization mode at 70 eV, scanning in the 30–600 *m*/*z* range. The ion source and transfer line were both kept at 280 °C. The collected data were processed by ChromSpace (SepSolve Analytical, UK), and the pyrolysis products were identified using the NIST library database.

### 2.2 Test Organisms

The earthworm, *Eisenia fetida,* was chosen for this study as it is a standard invertebrate species for ecotoxicological assessment in protocols by the OECD and the International Standardization Organisation.^35,36^*E. fetida* are also ecologically important detritivores; they feed on leaf litter and other sources of organic matter at the soil surface playing a vital role in decomposition and nutrient turnover.^37^*Eisenia fetida* was sourced from a commercial supplier (Blades Biological, Kent, UK), and held in synchronous culture at the University of Plymouth for at least 2 weeks prior to their use in experiments. Earthworms were kept in Lufa 2.2 soil with surplus horse manure (from unmedicated horses) as feed, at a temperature of 20 ± 1 °C on a 12 h: 12 h light: dark cycle. Adult, clitellate worms were hand selected for the experiments.

### 2.3 Lethal Toxicity and Establishing Sublethal Thresholds

There are sparse data on the lethal toxicity of biobased plastics to soil organisms, and this experiment was conducted to derive dose–response curves for mortality over 72 h based on OECD Technical Guidance (TG) 207 for acute toxicity to earthworms according to the filter paper contact test method.^35^ Prior to the experiment, earthworms were collected from the stock cultures and held on damp filter paper for 24 h at 20 ± 1 °C in the dark to void their gut contents. The filter paper was changed after 12 h to prevent coprophagy. An appropriate dilution series of the number concentration of each material in ultrapure water (100, 320, 1000, 3200, 10000 fibers per mL) was made. A Sedgewick Rafter counting chamber was used to enumerate fibers mL^–1^ in stocks made in deionized water,^38^ with adjustments made as required to achieve the desired concentration. Mass concentrations for each material are included in the Supporting Information (Figure S7 and Table S5). Then, 1 mL of the relevant fiber dispersion was placed onto a filter paper (Whatman, 90 mm) contained within a 90 mm glass Petri dish, equating to a range of 1.6–157.2 fibers cm^2^. One *Eisenia fetida* earthworm was introduced to each Petri dish (*n* = 10 dishes per treatment). In addition, a negative control (deionized water with no fibers) was used and a positive control of 3,4-dichloroaniline (3,4-DCA). 3,4-DCA is used as a reference chemical in ecotoxicity testing, and the lethal concentration (LC_50_) was determined by fitting a dose–response curve (Supporting Information Figure S8 and Table S6). A solution was prepared containing 192 μg mL^–1^ dissolved in deionized water, equivalent 3.00 μg cm^2^ on the filter paper. The positive control was used to ensure that earthworms were responsive to the test system. Dishes were incubated in the dark at 20 ± 1 °C, with mortality and worm biomass recorded at the end of the 72-h exposure period.^35^ An earthworm was considered dead if it failed to respond to a gentle mechanical touch on the front end. Data were analyzed after curve fitting (see below) to select a sublethal concentration for the next experiment (Section 2.4).

### 2.4 Chronic Effects on Growth, Reproduction, and Bioturbation Activity

The experimental design here followed a similar approach to the acute toxicity study above except that chronic effects were investigated. The protocol followed the method for the OECD TG 222 earthworm reproduction test^39^ with some additional end points, as we have previously done for particulate materials.^40^ A control test soil (no fibers) and a positive control (192 μg mL^–1^ 3,4-DCA) were included as before, and the test materials: Polyester, Lyocell and Viscose fibers (125–300 μm length), each at a concentration of 100 mg fibers kg^–1^ dry weight soil (0.01% w/w). This exposure concentration (100 mg fibers kg^–1^ = 0.1 mg fibers g^–1^ see Supporting Information Table S5 for an estimate of numerical concentration) is environmentally relevant as it is within the range reported in soils, for example, 2500 fibers kg^–1^^29^ and 7–17,600 microplastics kg^–1^ soil.^11,27,28^

Lufa 2.2, a sandy loam soil (LUFA Speyer, Germany) was used with the following composition (supplier’s information, mean ± SD, dry soil): pH, 5.5 ± 0.1 (measured in 0.01 M calcium chloride solution); organic carbon, 1.66 ± 0.60%; nitrogen content, 0.19 ± 0.06%; cation exchange capacity, 8.5 ± 1.8 mequiv 100 g^–1^. The water-holding capacity of the soil was measured in house as 34.2 g 100^–1^ d.w. Prior to use, the soil was sieved through a 2 mm mesh and air-dried at 25 °C for 2 days. Soil pH was measured prior to the start and at the end of the experiment (in a 1:2.5 soil:water slurry, using a glass combination electrode, Corning 420).

Soil was dry dosed with fibers as this technique has been shown to be effective for particulate contaminants.^40−42^ A single batch of soil was dosed for each of the materials, followed by dividing it into eight replicates. Briefly, the mass of fibers required to dose the replicates (480 mg) was added to 50 g d.w. of soil and thoroughly mixed by hand for 10 min. A further 750 g dw of soil was added, and it was mixed again thoroughly, before adding the remaining soil (4000 g) and thoroughly mixing again. This process was undertaken to ensure an even distribution of fibers in the soil. Each replicate containing 600 g of the fiber-dosed soil was then wetted to 60% water holding capacity with ultrapure Milli-Q water (18.2 Ω) according to OECD^39^ and left to equilibrate for 2 days prior to the introduction of worms.

Adult *E. fetida* with a typical mean starting wet weight of 0.73 ± 0.08 (mean ± SEM, for a subsample of 12 earthworms) and fully developed clitellum were exposed in 4 replicate vessels measuring 25.4 cm × 34.6 cm x 19.2 cm (*n* = 12 earthworms per container, 48 per treatment) at 20 ± 1 °C in the dark. Horse manure (5 g from unmedicated horses), and 7 mL of deionized water were added weekly to each test container to provide nutrition.^40,42^ Survival and body weight were recorded at day 0 (start), 7 and 28 of the experiment. Earthworms were collected at days 7 and 28 for biochemistry and histopathology (details below). Reproductive output was determined by the number of egg cocoons produced at 28-days. Egg cocoons were returned to test containers and incubated for a further 28-days, whereupon the number of juveniles and their mass were quantified following OECD TG 222.^39^ In addition, near-daily observation through the 28-day experiment enabled the number of earthworm casts, i.e., a mound of soil displaced by the burrowing earthworm to the soil surface, to be recorded as a proxy for bioturbation activity.

#### 2.4.1 Trace Element Analysis of Earthworm Tissue

One of the primary modes of chemical toxicity in soft-bodied invertebrates is osmoregulatory disturbances, which can be assessed through the analysis of trace elements and electrolytes. Particulate contaminants, such as engineered nanomaterials, and microplastics have been reported to alter osmoregulatory function and metabolism.^43,44^ Trace-metal analysis was undertaken following the protocol outlined in Tatsi, Shaw, Hutchinson, and Handy.^40^ Three earthworms were sampled at random from each test container (*n* = 12 per treatment sampled at 7 and 28 days) and were depurated for 24 h on damp filter paper; the filter paper was changed after 12 h to prevent coprophagy. The earthworms were washed in deionized water and dried before being snap frozen in liquid nitrogen and stored at −80 °C until required. Earthworms were individually freeze-dried for 48 h, the dry weight was recorded, and the worms were acid digested at 70 °C with 70% analytical grade nitric acid for 2 h. The digests were allowed to cool, diluted to 20% with ultrapure water (18.2 Ω) and stored in the dark. To examine treatment-specific changes in the trace element and electrolyte composition of the earthworms, the essential elements Ca, Fe, K, Mg, Mn, Na, Zn were analyzed by ICP-OES (iCAP 700).

#### 2.4.2 Oxidative Stress

Whole earthworm tissue from samples collected at the end of the experiment were analyzed for oxidative stress (total glutathione) following the method outlined by Tatsi, Shaw, Hutchinson, and Handy.^40^ Two earthworms from each test container (*n* = 8 per treatment sampled at 7 and 28 days) were snap frozen in liquid nitrogen and stored at–80 °C until required. The earthworm tissues were diluted (1:5 ratio, weight: volume) in ice-cold isotonic buffer (150 mM sucrose, 50 mM HEPES, 1 mM EDTA, pH 7.3) and homogenized on ice (3 × 10 s with 2 min rests at 17,500 rpm, Cat X520D with a T6 shaft, Bennett Scientific Ltd., UK). Homogenates were centrifuged at 6000 rpm for 3.5 min to remove debris, and 1 mL aliquots of the supernatant were stored at −80 °C until required. Subsequently, aliquots of the crude homogenates were further diluted in the cold isotonic buffer (recipe above, 1:15 dilution, i.e., an overall 20-fold dilution of the original tissue) due to the high protein concentration of the earthworms. The diluted homogenates (20 μL) were assayed in triplicate for total glutathione (GSH), by measuring the change in absorbance at 4 s intervals over 10 min at 412 nm (see Supporting Information section S7 for detailed method)^45^ using a Spectramax Plus 384 plate reader (Molecular Devices, UK). The concentrations of GSH were normalized to total protein (25 μL of homogenate, Pierce BCA kit, Thermo Scientific, UK), and data are expressed as nmol GSH per mg protein (further details in Supporting Information sections S7 and S8).

#### 2.4.3 Histopathology

Histology was conducted as described in ref (46). Five earthworms per treatment were randomly chosen for routine histology. Worms were depurated for 24 h and fixed in buffered formal saline (10%). Transverse sections were cut through the clitellum and at three segments anterior to the clitellum and processed into paraffin wax blocks (Leica TP1020 tissue auto-processor and Leica HistoCore Arcadia H embedder) as described in Handy, Runnalls and Russell.^47^ For routine histological examination, 5 μm sections were cut from each earthworm block (Leica RM2235 microtome), then stained in batches with hematoxylin and eosin to show general architecture which was visualized using a Leica DMD 108 digital microimaging system. The presence and absence of pathology, such as necrotic cells, lipofuscin, and vacuole formation in the muscles were also noted.

### 2.5 Statistical Analysis

For both experiments, the 3,4-DCA positive control was used only to confirm the responsiveness of the test systems and was not included in statistical analyses. To estimate acute toxicity and effect thresholds, the cumulative mortality was calculated across the mass concentration and number concentration series for each fiber. Sigmaplot V14.5 was used to produce dose–response curves based on sigmoidal equations. The model fit was examined by the *r*^2^ values and the equation which best explained the curve variance (3-parameter or 4-paramter sigmoid) was selected. Lethal concentration estimates of LC_10_, LC_20_, and LC_50_ and no observed effect concentration (NOEC) for cumulative mortality were computed.

To analyze treatment-specific differences across biological end points from the chronic exposure experiment, the proportional fresh mass change of worms per container were calculated to account for individual variability. Reproduction data were also treated this way to derive the number of juveniles normalized by the number of egg cocoons, and the mass per juvenile normalized by the number which hatched. The cumulative number of casts produced on the soil surface was used as a proxy for bioturbation activity; quantities were grouped by experimental week for further analysis. Analyses were performed in RStudio 2022.07.1, with data tested for normality and equality of variance using Shapiro and Levene’s tests, respectively. Treatment and time effects on (a) the proportional mass change of worms, (b) bioturbation activity, (c) the concentration of total glutathione (nmol per mg of protein), and (d) tissue elemental composition, were determined using 2-way Analysis of Variance (ANOVA), and treatment only effects were analyzed by one-way ANOVA where appropriate. In all cases, Tukey’s honest significance difference (HSD) was used to analyze pairwise differences.

## 3 Results and Discussion

### 3.1 Chemical Characterization of the Fibers

The FTIR analysis confirmed the chemical composition of the selected textiles. Polyester samples present typical absorption bands of its constituent polymer, poly(ethylene terephthalate) (PET), that are CH_2_ stretching (2961–2855 cm^–1^), C=O stretching (1712 cm^–1^), C=C aromatic stretching (1504 cm^–1^), C–O esteric stretching (1240 and 1092 cm^–1^), aromatic ring in-plane CH bending (1016 cm^–1^) and in-phase CH wagging (721 cm^–1^)^48^ (see Supporting Information section S2). The FTIR spectra of viscose and lyocell samples are almost identical and present the same absorption bands characteristic of cellulose-based polymers: O–H stretching at 3309–3310 cm^–1^, signal at 1638 cm^–1^ due to the vibration of the OH of the absorbed water molecules, −OH in plane bending at 1333–1335 and 1199–1200 cm^–1^, C–O–C asymmetric stretching at 1155 cm^–1^ (see Supporting Information section S2 for the full list of assigned absorption bands^49^). Powder X-ray diffraction (Supporting Information section S4) indicated that viscose fibers were less crystalline than lyocell fibers, meaning that the polymeric chains have a less organized structure which in turn affects the material’s water retention capacity.

A more in-depth characterization of the textile samples was performed through Py-GCxGC-TOF MS, and results are in line with those previously reported^50^ (Supporting Information section S3). The pyrogram of the polyester samples present mostly aromatic species, such as terephthalates, and PET pyrolysis markers like vinyl benzoate.^51^ The pyrolysis products of viscose and lyocell samples include mainly aldehydes, ketones, furans and anhydrosugars (like levoglucosan and 1,4:3,6-dianhydro-α-d-glucopyranose) with their derivatives, typical compounds of cellulose-based materials.^52^ Besides minor differences in the compounds identified, it was not possible to detect specific markers that enable discrimination between the two biobased fibers. Py-GCxGC-TOF MS data did not detect the presence of additives, nonintentionally added substances (NIAS) or finishing products in any of the materials studied.

### 3.2 Acute Toxicity and Effect Thresholds

**Figure 1:**
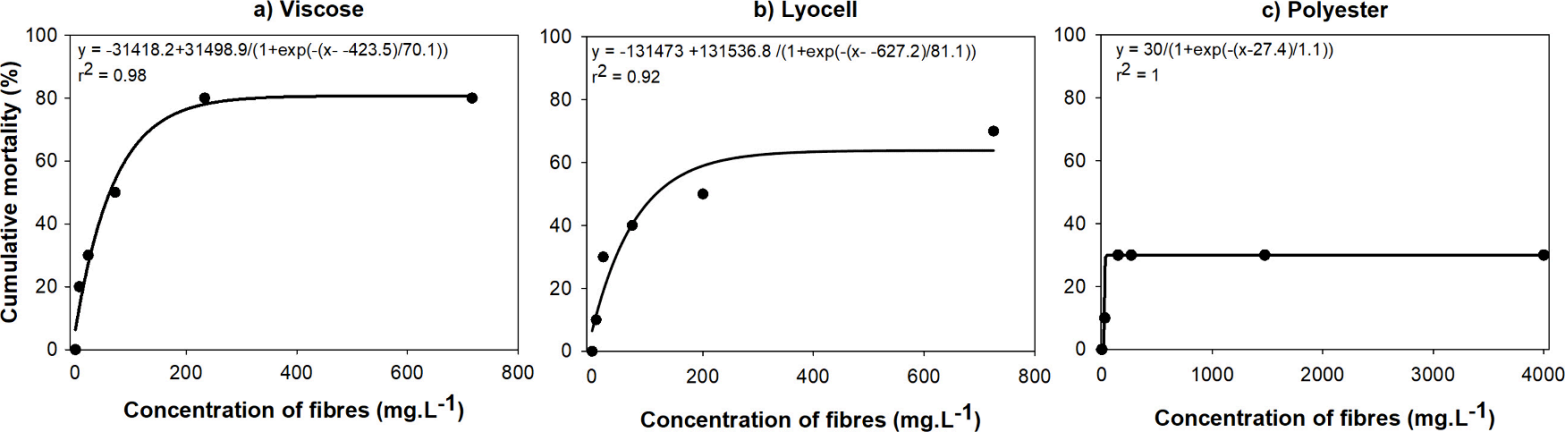
Dose–response plots of the mean cumulative mortality, as a percentage, of *Eisenia fetida* (*n* = 10) following 72-h exposure to a concentration series of (a) viscose, (b) lyocell, and (c) polyester fibers (mg·L^–1^). Equations and *r*^2^ values are shown on each plot. Note: The standard error could not be derived due to the single value for worm population mortality at each fiber concentration.

Overall, the dose–response (Figure 1) and the lethality estimates (Table 2) indicate that the biobased polymers cause greater mortality of *E. fetida* than conventional polyester. For polyester, despite increasing concentrations to 1000 mg·L^–1^, only around 30% mortality was detected. In contrast, the cumulative LC_50_ values for viscose and lyocell were 61.59 and 116.11 mg·L^–1^, respectively, with viscose being the most toxic. However, at lower concentrations, the LC_10_ and LC_20_ values for lyocell and viscose were similar (Table 2). The calculated no observed effect concentrations (NOECs) for mortality were within the range 0–2.8 mg·L^–1^ for both viscose and lyocell, while for polyester fibers the NOEC was in the range 0–15.6 mg·L^–1^ (Table 2), illustrating the higher toxicity of the biobased fibers. While lyocell is sometimes presented as a more environmentally friendly alternative to viscose due to its production processes,^30,53^ this was not evident and the comparative toxicity of these materials is supported by their chemical similarity (Section 3.1). As noted above, fibers did not have associated chemical additives; therefore, it is likely that toxicity was influenced by the particle and aspects of its physical characteristics, rather than by concomitant chemicals.

**Table 2:** Estimated Effect Thresholds Inducing 10%, 20%, and 50% Mortality in *E. fetida* and No Observed Effect Concentration (NOEC), Presented As Fiber Number and Mass Concentrations Following 72-h Exposure^a^

	By particle number concentration (fibers mL^–1^)				By mass concentration (mg·L^–1^)			
Material	LC_10_	LC_20_	LC_50_	NOEC	LC_10_	LC_20_	LC_50_	NOEC
Viscose	39.06	195.31	859.38	0–39	2.80	14.00	61.59	0–2.8
Lyocell	113.19	273.44	1406.25	0–39	5.66	22.66	116.11	0–2.8
Polyester	78.1–117.2	78.1–117.2	b	0–78	0–15.6	15.6–31.3	b	0–15.6

a The standard error could not be derived due to the single value for worm population mortality at each fiber concentration.

b LC_50_ for polyester could not be calculated as mortality >30% was not achieved.

The most appropriate metrics for reporting toxicity data for plastic particles (e.g., mass concentration, particle number concentration, per unit surface area, per fiber length, etc.) is still being investigated.^54^ In regulatory ecotoxicity testing and under regulations such as the Registration, Evaluation, Authorisation and Restriction of Chemicals (REACH) in the EU, mass concentration is generally used (e.g., mg L^–1^), but environmental studies in the scientific literature are often presented as particle or fiber number concentrations (i.e., counts mL^–1^). To enable comparison with other studies, fiber number concentrations are also shown here (Table 2). Inevitably, an acute dose–response curve may cover a wide range of concentrations; the fiber number concentrations are environmentally pertinent at the lower range of concentrations, while at the higher limits, the concentration of fibers exceeds those typically reported in agricultural soils. For example, between 7–6,360 microplastics (MP) kg^–1^ have been reported where there has been no history of biosolid application to soil,^27,28^ and 97–17,600 MP kg^–1^ where biosolid amendments had been applied.^11^ Indeed, sewage biosolids can introduce substantial quantities of microplastics to soils, a review highlighted that globally biosolids can contain between 0.2–16,9000 microplastics/g, of which the majority are reported as fibers.^11^ There seem to be no specific reports on the proportions of viscose and lyocell fibers in agricultural soils, so far, but the estimated LC_50_ values of 859.38 and 1406.25 fibers mL^–1^, respectively (Table 2), are well below the total plastic particle/fiber counts noted above in natural soils. Further work is required to determine the environmental degradation rate of regenerated cellulose fibers to estimate their environmental persistence. However, a further challenge is also a lack of routine detection methods to differentiate biobased polymers from other materials present in soils.

### 3.3 Material-Specific Effects on Adult Survival, Growth, and Bioturbation Activity

Exposure to 0.01% w/w fibers had no significant effect on the survival of adult earthworms. After 7 days, only 1 individual had perished from one of the control containers. No mortality was recorded following exposure to viscose or polyester at either time point (7-days and 28-days). After 28 days of exposure to lyocell, three worms had perished (each from different containers), resulting in 6% total mortality. These results are as expected based on the calculated lethal concentrations (Section 3.2) and are in line with previous studies which find low levels of earthworm mortality following soil exposure to microplastics/fibers.^31,55^

**Figure 2:**
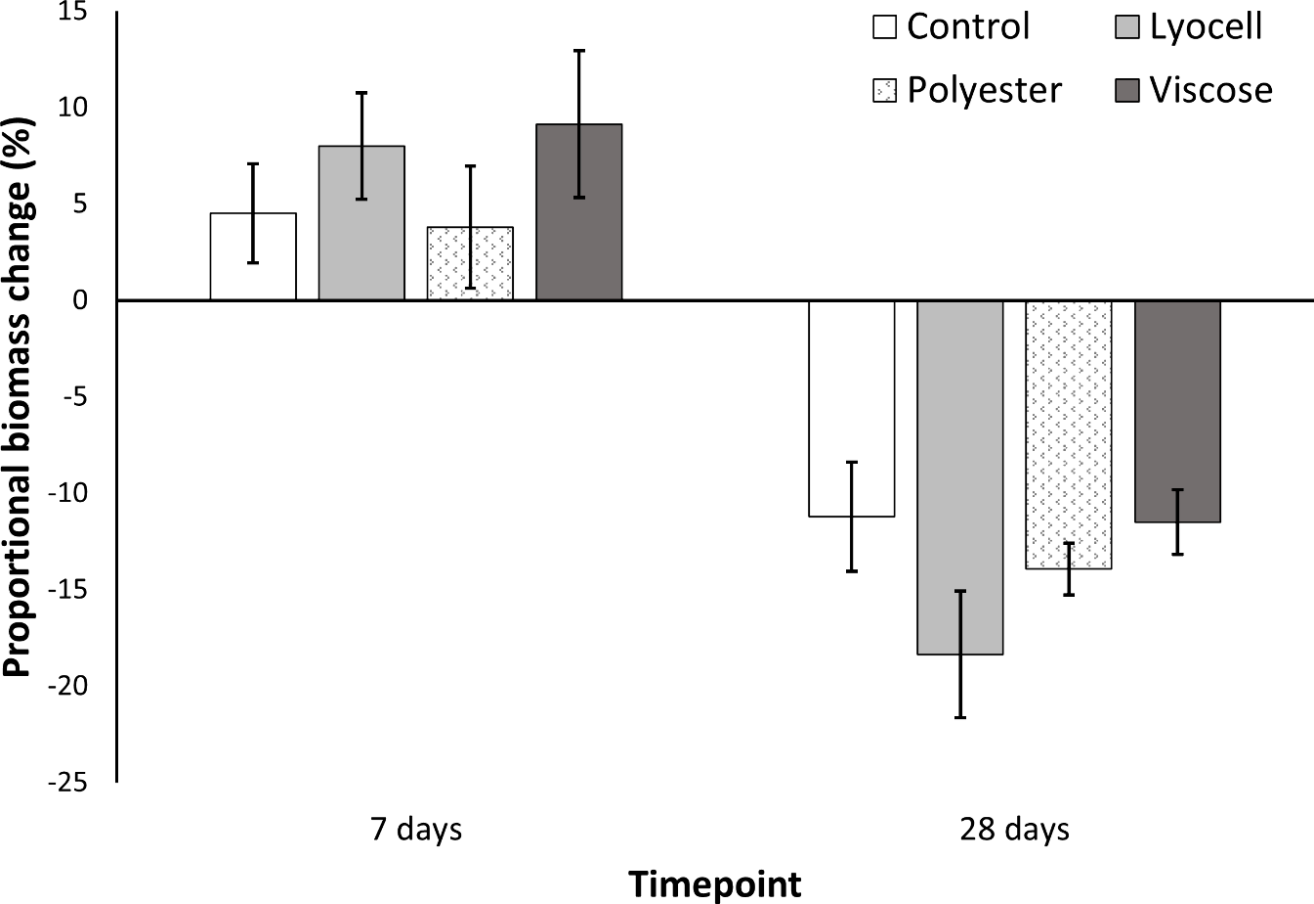
Mean proportional biomass of *E. fetida* across treatment groups after 7 and 28 days (*n* = 4). Error bars show SEM, and there were no statistical differences identified between groups.

**Figure 3:**
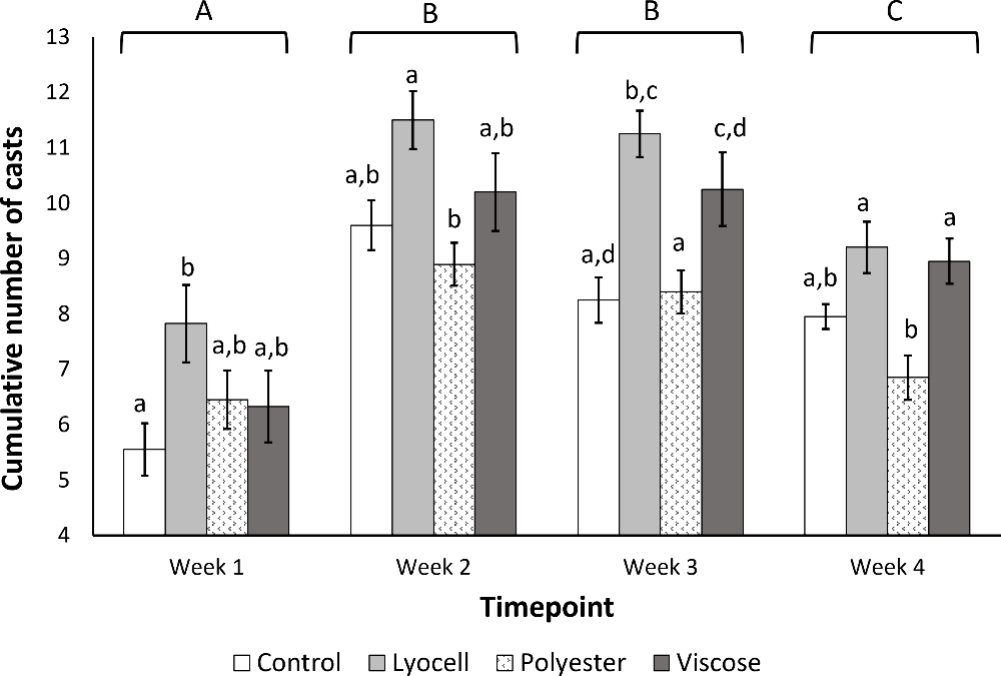
Cumulative number of casts produced by *E. fetida*, grouped by week, indicative of bioturbation activity. Data show mean (week 1: *n* = 8 replicates; weeks 2–4: *n* = 4 replicates) ± SEM, different lowercase letters show significance between treatment groups within weeks, while uppercase letters show differences between week groups.

With respect to growth, the average wet weight of worms per replicate container increased in all treatments after 7 days, while it decreased after 28 days (Figure 2). Growth was most severely impacted in the lyocell (−18 ± 3%) > polyester (−14 ± 1%) > viscose (−12 ± 2%) > control (−11 ± 3%) treatments; however, material-specific differences on growth were not statistically significant after 7-days (*F*_(3,12)_ = 0.702, *p* = 0.569), nor after 28-days (*F*_(3,12)_ = 1.857, *p* = 0.191). A two-way ANOVA indicated that experiment duration, rather than treatment, or an interaction between these factors, was the main variable explaining the mass changes in worms (*F*_(1,24)_ = 104.09, *p* < 0.001). Worms were supplied with adequate manure throughout the exposure, thus nutrient deficiency does not explain this pattern. However, the activity of the earthworms may offer an explanation. Bioturbation activity varied significantly between the exposure material (*F*_(3,383)_ = 12.365, *p* < 0.001) and time point (grouped into weeks; *F*_(3,383)_ = 34.166, *p* < 0.001), with the main effect attributed to time point. Adult *E. fetida* had significantly lower bioturbation activity within the first week compared to all other time points (*p* < 0.001; Figure 3) and therefore were able to allocate more energy to growth within this first week, while as activity increased in weeks 2 and 3 less energy was available for growth. Clearly, the overall bioenergetics of the organism are important, and there may be a trade-off between growth and locomotion during the present study.

Only one other study^18^ has examined the effect of microplastics on earthworm activity, and this showed a similar pattern where the growth of *Lumbricus terrestris* was suppressed in polyethylene (PE) microplastic treatments when bioturbation activity was high. We also noted some material-specific differences, where bioturbation activity was, in many cases, significantly higher in the viscose and/or lyocell treatments than in the polyester or control groups (Figure 3). The number of casts did not vary between control and polyester treatments, which aligns with observations for *L. terrestris* exposed to 7% w/w PE microplastics,^18^ quantities far exceeding those used in the present study. The process of bioturbation has been shown to redistribute microplastics within soils.^56,57^ The increased bioturbation activity exhibited by *E. fetida* in response to biobased fibers could enhance their transport through the soil profile, where organisms which inhabit deeper layers of soil could interact with the plastic particles. To our knowledge, this is the first study investigating the bioturbation activity of earthworms in response to biobased fibers, and more research is therefore required.

### 3.4 Sublethal Effects on Reproductive Output

**Figure 4:**
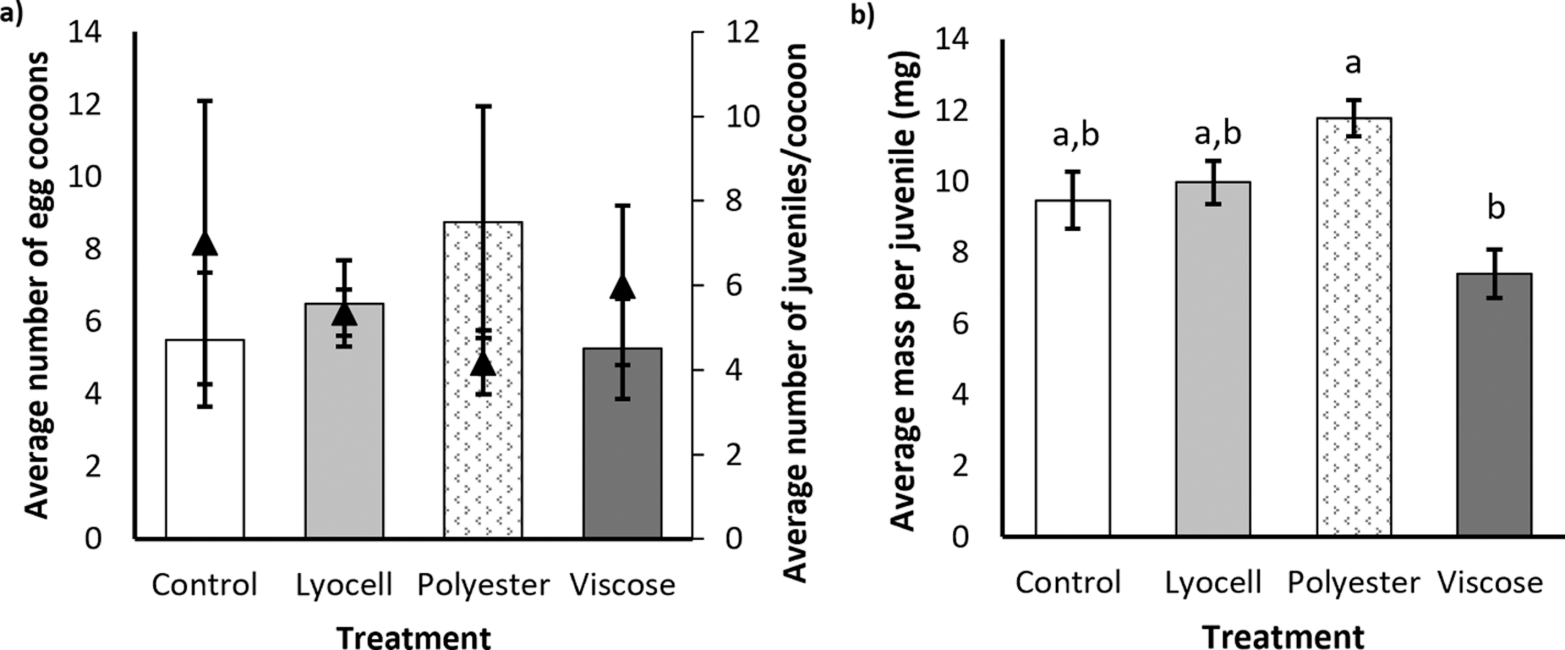
Reproductive output of *E. fetida* in terms of (a) the mean number of egg cocoons (bars) and the mean number of juveniles per cocoon (shown by the triangles on the secondary axis) and (b) the average fresh mass per juvenile following a 28-day exposure to experimental treatments. Error bars show standard error of the mean (SEM), and different letters denote statistical significance between groups (*p* < 0.05), note: there are no statistical differences in panel a.

Reproductive assays are regarded as sensitive parameters to evaluate toxicity and are used widely in regulatory toxicology due to the implications that altered reproductive output has on the population. Over the 28-day study, there were marked material-specific differences on the average mass of juvenile earthworms, however the number of egg cocoons (*F*_3,12_ = 0.549, *p* = 0.658; Figure 4a) and the normalized number of juveniles emerging from those cocoons (*F*_3,12_ = 0.295, *p* = 0.828; Figure 4a) remained relatively constant between treatments. Significant differences in the wet weight body mass per juvenile (*F*_3,12_= 7.472, *p* = 0.004; Figure 4b) were attributed to lower progeny mass in the viscose treatment compared to polyester (*p* = 0.002). Reduced offspring mass may impact future survivability and delay the onset of development and sexual maturity, which together can lead to changes in population dynamics.^58,59^ It is important to note that while the mass per juvenile was lower in the viscose treatment, it was not statistically different from the control.

One explanation for the differences between viscose and polyester could be due to the exposure doses. While earthworms were subjected to 100 mg kg^–1^, this equated to a lower measured particle number of polyester than of the biobased fibers (see Supporting Information section S5). However, if toxicity was only influenced by particle number, one would expect similar responses between *E. fetida* in the viscose and lyocell treatment (as their mass and number concentrations were comparable; Supporting Information Figure S7), which was not reflected in the results. Chemical characterization of the fibers, by Py-GCxGC-TOF MS, did not detect the presence of additives and the pyrolysis products identified for both lyocell and viscose are almost identical (Supporting Information section S3) showing the chemical similarity of these two types of fibers. Therefore, observed effects are likely due to aspects of the material’s physical/structural properties. Lower body mass (fresh weight) in juveniles exposed to viscose could be explained by a nutritional deficit and/or osmotic water loss. The juveniles were too small for any attempt at nutritional proximate analysis, but their small size (large surface area/volume) and less mature development of the epidermis, likely offers a risk of dehydration. This is normally prevented by the moisture content of the soil (held at 60% water holding capacity during the experiments), but interestingly, viscose is more hydroscopic than polyester or lyocell.^32,60^ While water regain values of materials can be broad,^32^ Siroka, Noisternig, Griesser, and Bechtold^60^ report that “unwashed” lyocell fibers have a water regain value of 39.27 mmol/g while for viscose this is measured as 48.62 mmol/g. This could further be influenced by the fibers’ surface structure; viscose fibers have longitudinal striations (Supplementary Figure S3) which can act to increase the surface area: volume ratio, compared to lyocell (where fibers are generally smooth; Supplementary Figure S2). Therefore, it is possible that viscose in direct contact with the epidermis might cause a local dehydration of that microenvironment, leading to osmotic stress. This hypothesis is supported by our crystallinity measurements, which show that viscose is less crystalline than lyocell (Supporting Information Figure S6). This means that the polymeric chains have a less organized structure which affects water retention and could partly explain the possible higher local dehydration in the viscose treatments (Figure 4b). Earthworms secrete mucus from glands to maintain gas exchange, lubricate movement and to protect against pathogenic microorganisms.^61^ Mucus is highly conserved across species, and the freshly secreted mucus typically requires more than 95% hydration to become physiologically functional.^62^ Those functions, including osmoregulation at the epithelial surface, could be lost if secreted mucus competes with polymers for hydration. The 125–300 μm fibers used would have been too large for the juvenile worms to ingest, and so an epidermal rather than nutritional effect seems more likely. Regardless, the interference of plastic polymers with biophysical processes at epithelial surfaces requires more investigation.

Polyester fibers had no discernible effects on the fecundity or juvenile quality compared to the control group. This result was somewhat surprising as literature has documented suppressed reproduction of *Daphnia magna*,^63^*Ceriodaphnia dubia*,^64^ and soil-dwelling *Enchytraeus crypticus*,^65^ following polyester fiber exposure. One explanation for the differing results could be that toxicity was largely driven by additives/colorants on the fibers,^66^ as reported for other plastic products.^67^ These studies^63−65^ all used shop bought clothing to generate fibers, while the fibers used in this study were obtained directly from the textile producer prior to the incorporation of chemical finishing products and dyes, and confirmed by chemical analyses.

### 3.5 Sublethal Effects on Organism Health

**Figure 5:**
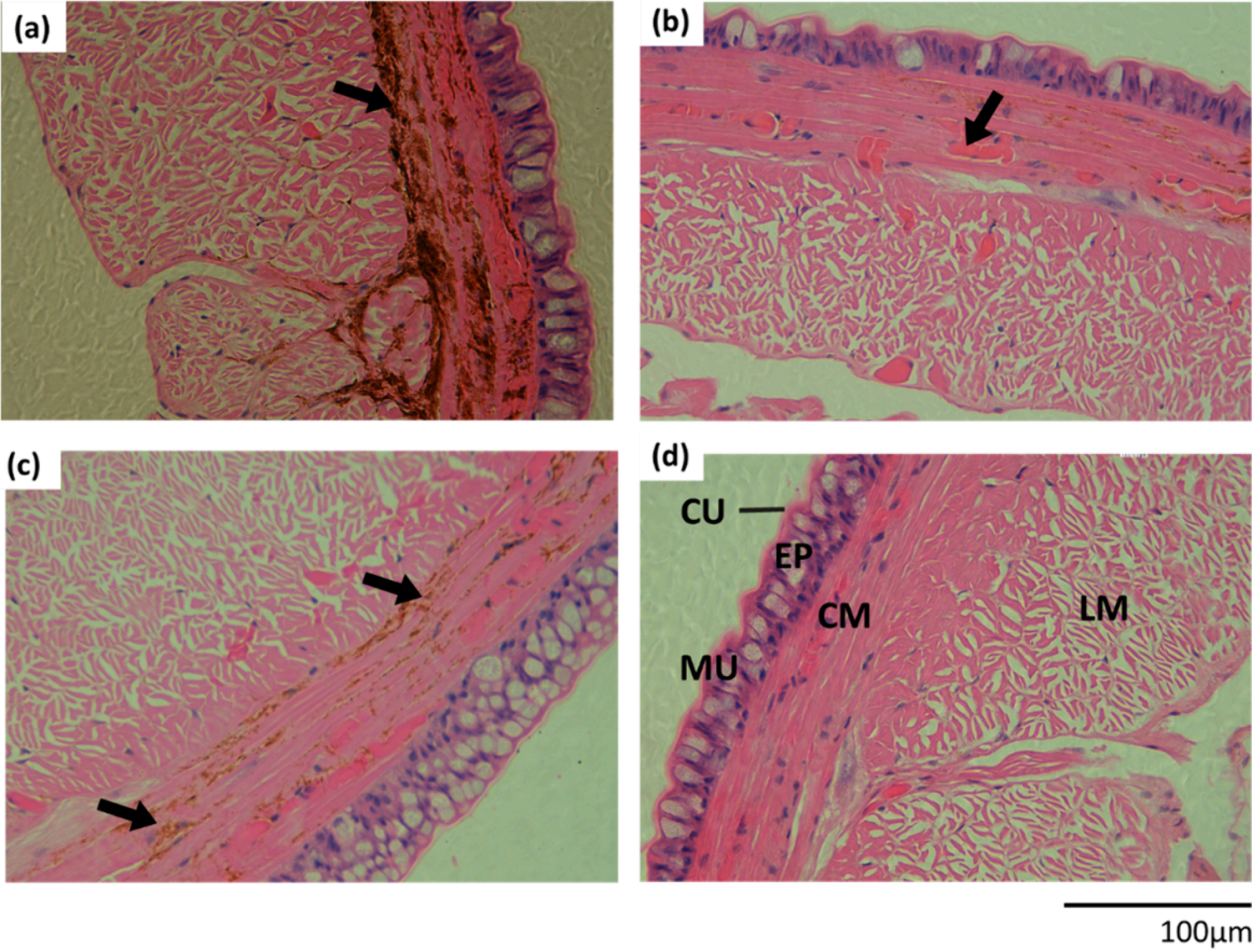
Example transverse sections of *Eisenia fetida* following 28-day soil exposure to (a) no fiber control group, and 100 mg kg^–1^ of (b) viscose, (c) polyester, and (d) lyocell fibers. CU, cuticle; EP, epidermis; CM, circular muscle; LM, longitudinal muscle; MU, mucous cells (clear cylindrical cells with slight deposits). Arrows point to pigmented regions of lipofuscin (tissue degradation), indicative of a stress response. Scale bars apply to all images.

The overall health of the earthworms were assessed histologically and with measurements of electrolyte concentrations (osmoregulation) and biochemistry relating to oxidative stress (total glutathione). Histopathological evaluation revealed that the cuticle and epidermis of the adult worms were intact (Figure 5). The epidermis showed the expected normal morphology, with the absence of necrosis, reactive hyperplasia, edema, or hydrophic change, and no evidence of inflammation and intact mucous cells. This indicates that the plastic fibers were not an irritating chemical to the skin, and the integrity of both the cuticle and epidermis suggests that uptake of intact plastic fibers through the skin was unlikely, with ingestion being the main route of exposure. Pathology was also generally absent from the circular and longitudinal muscle, with no evidence of fibrosis, hyperplasia or foci of necrosis. However, there were apparent differences in the presence and extent of lipofuscin deposits, especially in the circular muscle (Figure 5), and this was more severe in the lyocell > polyester > viscose > control treatments. Lipofuscin is a byproduct of the normal process of cell turner and degeneration through the lysosomal pathway, and contains oxidized lipids and proteins.^68^ In this study, the surrounding tissues were reasonably healthy (Figure 5), and the increased lipofuscin deposits are therefore interpreted as increased efforts in tissue repair and maintenance. This tissue repair must have been successful, because there were no statistical differences in the concentration of total glutathione (GSH), a primary antioxidant defense, between treatment groups or between exposure times (7 and 28 days; Supporting Information Figure S9). This concurs with Rodriguez-Seijo, da Costa, Rocha-Santos, Duarte and Pereira,^31^ who reported an up-regulation in GSH, along with other stress-related biomarkers in *E. fetida* only at far higher microplastics exposure scenarios (>500 mg kg^–1^ soil) than used in the present study.

**Figure 6:**
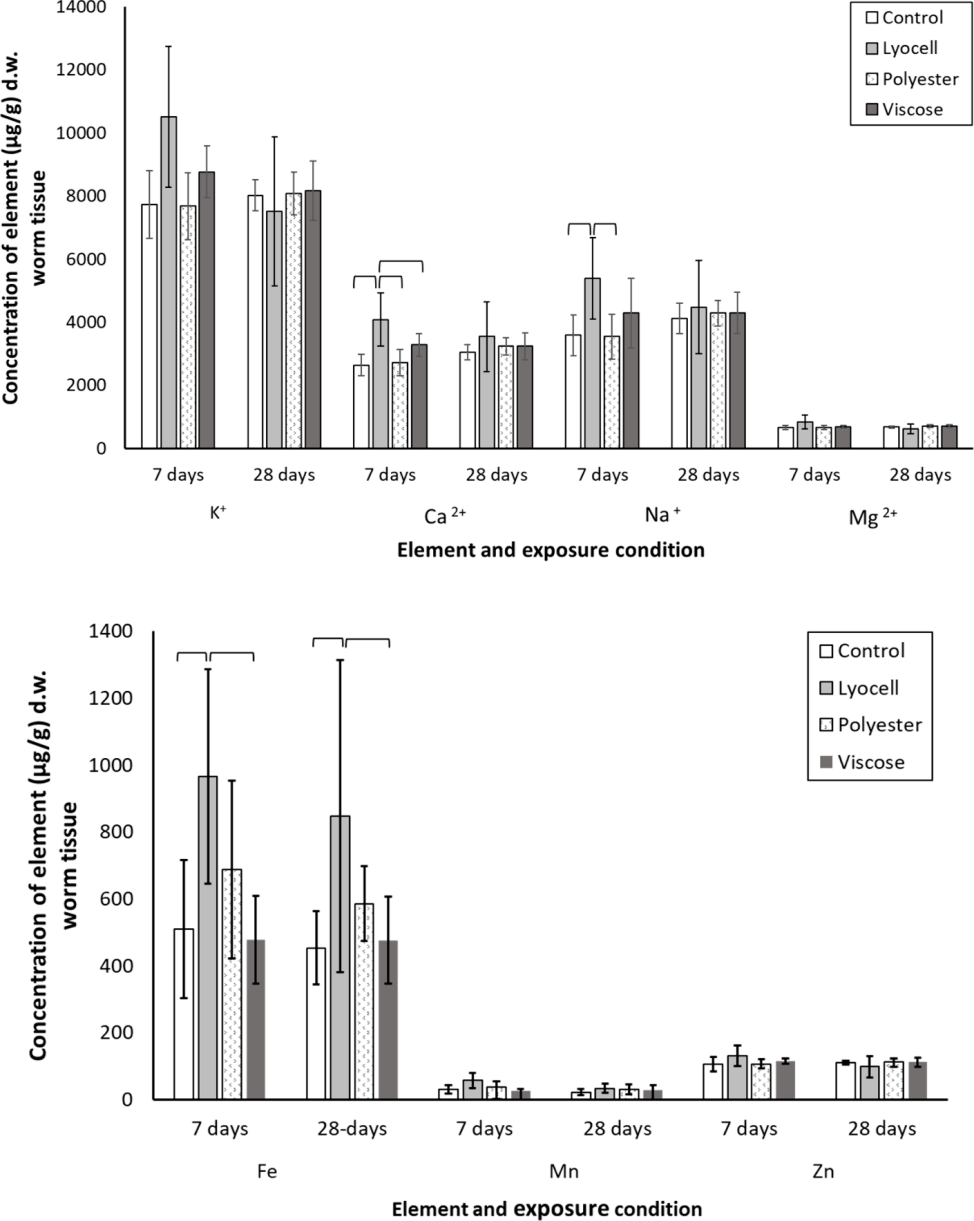
Concentration of (a) electrolytes (K^+^, Ca^2+^, Na^+^, Mg^2+^) and (b) trace metals (Fe, Mn, Zn) per gram dry weight of *E. fetida* tissue following 7- and 28-days exposure to fibers. Data presented are mean values (*n* = 12 individuals/treatment/time) ± standard deviation; parentheses illustrate significant pairwise differences between treatment groups.

Determination of electrolyte (Ca^2+^, Mg^2+^, K^+^, Na^+^) and trace element (Mn, Fe, and Zn) composition was carried out as previous studies indicate that particulate contaminants, including microplastics, cause trace element disturbances in earthworms; for example, polystyrene microplastics altered the osmoregulatory metabolism of earthworms.^44,69^ Furthermore, microplastics have been shown to adsorb trace metals^70^ potentially altering their bioaccessibility in soils. The compositions of tissue electrolytes and trace elements are shown in Figure 6. There were no wholesale increases or decreases in all electrolytes, indicating that general osmotic stress did not occur. However, there were some electrolyte-specific and material-specific changes. Overall lyocell exposed earthworms had the greatest variation in tissue electrolyte composition compared to the other treatment groups. The concentration of calcium (Ca^2+^) and manganese (Mn) were both significantly elevated following 7-day exposure to lyocell compared to polyester, viscose or the control group (Ca^2+^: *p* < 0.0001, *p* = 0.004, *p* < 0.0001; Mn: *p* = 0.0209, *p* = 0.0001, *p* = 0.0008, respectively). Similarly, the sodium (Na^+^) concentrations were higher following 7-day exposure to lyocell than to polyester (*p* = 0.0002 and *p* = 0.0209) or compared to the control (*p* = 0.0003). A mechanistic explanation from the viewpoint of ion transporters is unclear, when increases in both Ca^2+^ and Na^+^ occur without changes in K^+^ (e.g., not increased Na^+^ uptake on the Na^+^/Ca^2+^ exchanger or via Na^+^K^+^2Cl^–^ symport^71^). An increase in Mn is interesting, as this is required for the respiratory burst in immune cells,^72^ and may also be deposited in lipofuscin.^68^ Regardless, the increases in ion concentrations were transient, with no treatment-dependent differences by 28-days. In contrast, the treatment-specific increases of iron concentrations were sustained throughout the experiment duration, with earthworms in the lyocell treatments showing higher Fe than the viscose (*p* = 0.0001, *p* = 0.0035) or the control group (*p* = 0.0002, *p* = 0.0022) after 7 and 28-days, respectively. Iron is in excess in the environment, and dietary uptake of iron is only usually stimulated in deficiency, so it is unclear why the total iron concentrations increased in the tissue. Notably, iron catalyzes the Haber–Weiss reaction for free radical formation and the synthesis of lipofuscin. The elevated iron may therefore contribute to the appearance of lipofuscin deposits in the tissue (Figure 5). Through the experiment the concentration of magnesium and zinc did not significantly change and are broadly similar to values reported for earthworms.^40^

### 3.6 Study Implications

A key concern for the environmental risk assessment of biobased plastics is whether they present a greater hazard than conventional polymers used in equivalent applications. Lethality estimates (LC_*x*_ values and NOECs) are used under the Registration, Evaluation, Authorisation and Restriction of Chemicals (REACH) regulations to inform hazard evaluation.^73^ Based on lethality estimates, this study demonstrates that the biobased fibers studied (viscose and lyocell) induce greater mortality of *E. fetida* and thus are more hazardous, than polyester fibers. Additionally, across a range of sublethal end points selected for their relevancy determining individual and population-level effects, the biobased fibers induced greater effects than polyester. The comparable, if not more potent, toxicity of biobased materials has now been documented on a range of organisms (lyocell fibers on *D. magna*;^74^ poly(lactic acid) particles on *E. fetida*(23)) including the present study with *E. fetida*. Material-specific responses were also apparent for sublethal end points. The potential for suppressed reproduction was observed when worms were exposed to environmentally relevant concentrations (100 mg kg^–1^ soil ≡ 0.01%)^11,27−29^ of viscose fibers, compared to polyester. Increased bioturbation activity and a reduction in worm biomass was identified where soils were contaminated with lyocell fibers, which can alter ecological processes such as nutrient cycling.^16,17^ Chemical characterization (Py-GCxGC-TOF MS) of the materials did not detect the presence of additives, NIAS or finishing products, which could have influenced toxicity,^67,75,76^ and show a chemical similarity between viscose and lyocell. Therefore, it is likely the particles and aspects of their physical and inherent structural properties that influenced the toxicity observed.

The production of biobased fibers are projected to increase in the coming decades,^3^ with their use in the textile and apparel, medical, and automotive sectors, among others.^7,77,78^ This study contributes much needed data on the acute and chronic effects of biobased fibers, but further data are needed on other species in order to construct the species sensitivity distributions used in risk analysis, and with data from real exposure scenarios to estimate the probable risks and uncertainties. Unlike the regulations for medicines, in the environmental sector, there is no statutory requirement during registration in the UK, USA or EU to show that the new substance is safer or more effective than an existing traditional product. Instead, iterative steps of ecological risk assessment and risk management on the ground are needed to ensure responsible innovation with biobased polymers promoted as plastic alternatives and substitutes under the UN Plastics Treaty.

## Data Availability

Data are available: 10.5285/577486c2-c6e1-4588-957d-69c1a3d85628.

## Supporting Information Available

Supplementary characterization methods and results of fibers (S1–S4); fiber concentrations (S5); and supplementary methods and results of toxicity assessments (S6–S8) (PDF)
