# Supplementary material for: Are Biobased Microfibers Less Harmful than Conventional Plastic Microfibers: Evidence from Earthworms
Source: Environ Sci Technol. 2024 Nov 5;58(46):20366–77. doi: 10.1021/acs.est.4c05856 (PMC11580163; doi:10.1021/acs.est.4c05856)
Supplement: Supplementary file 1 — es4c05856_si_001.pdf [file es4c05856_si_001.pdf]

Are biobased microfibres less harmful than conventional plastic microfibres: Evidence from earthworms

W. Courtene-Jones, [1,2*] F. De Falco, [1,3] F. Burgevin, [4] R. D. Handy[1], R. C. Thompson [1]

[1] School of Biological and Marine Sciences, University of Plymouth, Drake Circus, Plymouth, Devon PL4 8AA, UK

[2] School of Ocean Science, Bangor University, Anglesey LL59 5AB, UK

[3] School of Geography, Earth and Environmental Sciences, University of Plymouth, Drake Circus, Plymouth, Devon PL4 8AA, UK

[4] Institute for Sustainability, Department of Chemistry, University of Bath, Bath BA2 7AY, U.K

* Corresponding author: w.courtenejones@bangor.ac.uk

Supplementary information

Summary: This file contains 14 pages, 9 figures, 5 tables

Contents

S1. Scanning electron imaging 1
S2. Fourier Transform Infrared spectroscopy 3
S3. Characterisation of textile fibres by Py-GCxGC-TOF MS 4
S4. Powder X-ray diffraction 9
S5. Fibre enumeration 10
S6. Lethal toxicity of 3,4-Dichloroaniline 11
S7. Supplementary method for determination of total glutathione concentrations 12
S8. Results: Oxidative stress 12
S9. References 13

S1. Scanning electron imaging

Morphological analysis of the textile fibres was performed by scanning electron microscopy (SEM) using a JEOL 6610 LV SEM (Tokyo, Japan). Samples were placed on stubs with adhesive tape and sputter-coated with gold prior to analysis. SEM observations were performed in high

vacuum mode, with an accelerating voltage of 15 kV. Scanning electron micrographs of the textiles fibres: polyester (Figure S1), viscose (Figure S2) and lyocell (Figure S3) are shown.

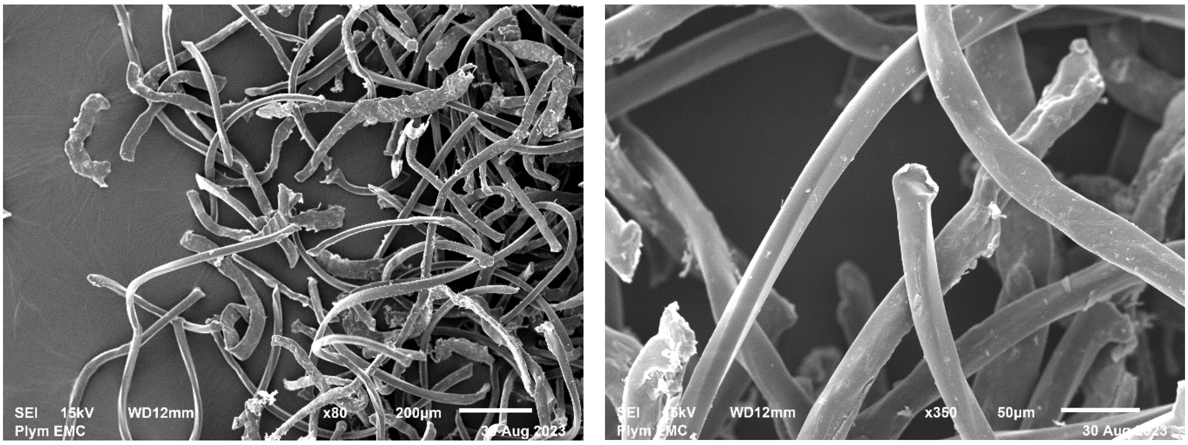

Figure S1. Scanning electron micrographs of polyester fibres used in this study at x80 and x350 magnification.

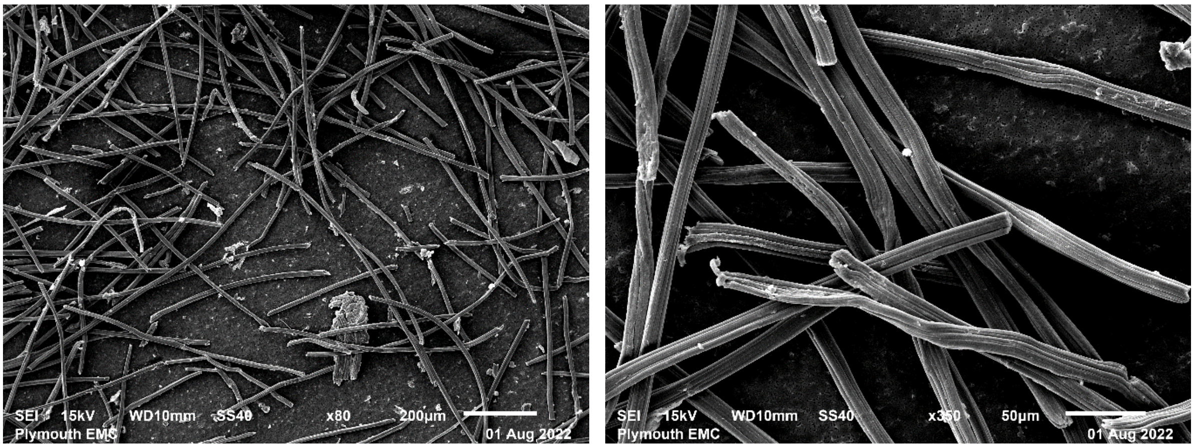

Figure S2. Scanning electron micrographs of viscose fibres at x80 and x350 magnification. Characteristic longitudinal striations can be observed on the viscose fibres.

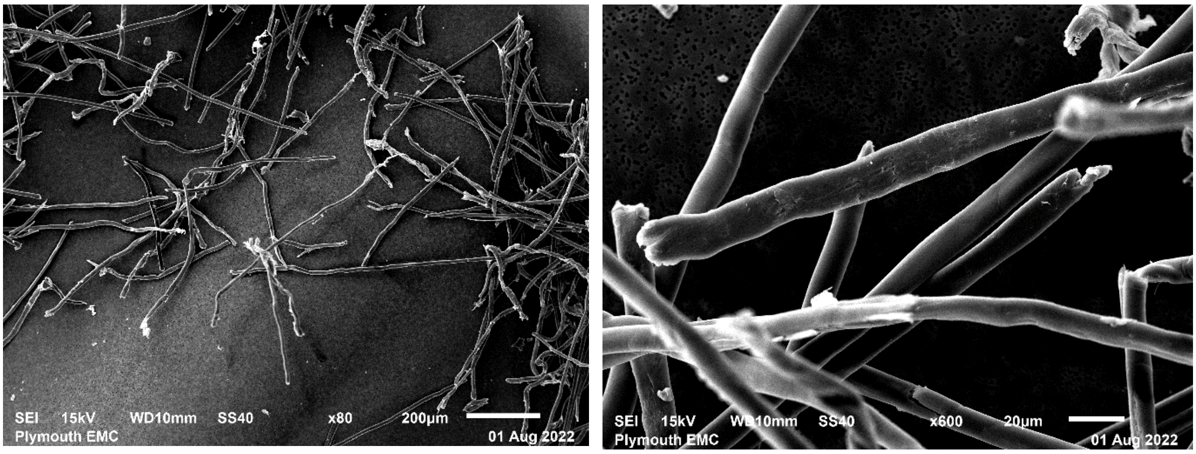

Figure S3. Scanning electron micrographs of Lyocell fibres at x 80 and x600 magnification.

S2. Fourier Transform Infrared spectroscopy

Attenuated total reflectance Fourier transform infrared (ATR-FTIR) spectroscopy was used to characterise the textile fibres. Spectra were acquired using 32 scans and a resolution of 4 cm^{-1}, over the range 4000–400 cm^{-1}. The resulting spectra (Figure S4) along with the vibrational mode assignment table (Table S1) are presented.

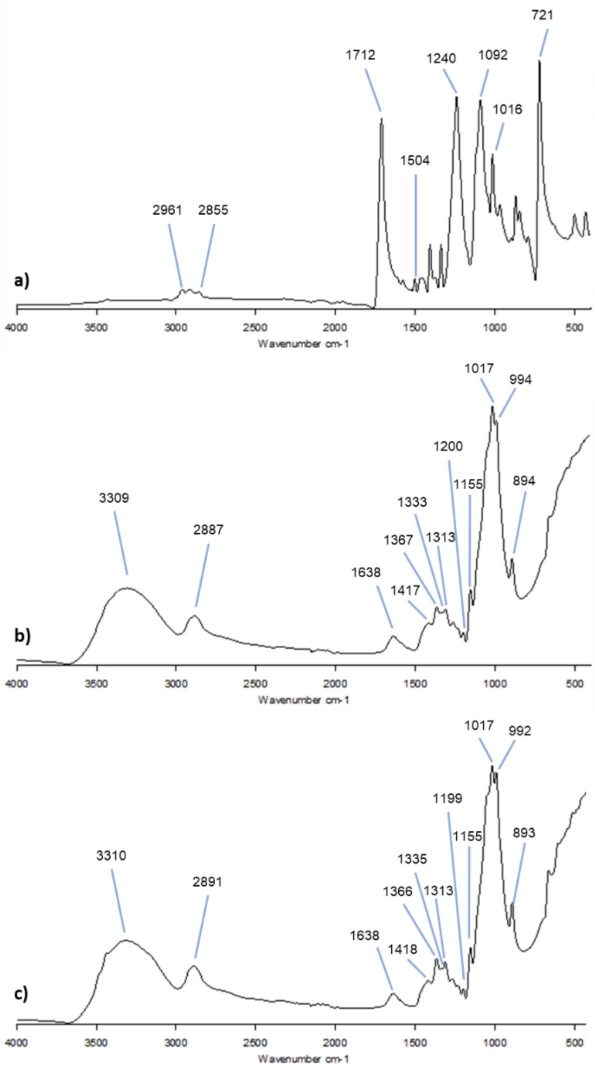

Figure S4 . ATR-FTIR spectra obtained for the samples composed of a) polyester, b) viscose and c) lyocell. Band assignments are reported in Table S1.

Table S1. Vibrational mode assignment for the ATR-FTIR spectra of the samples. Data are based on Noda, Dowrey, Haynes and Marcott [1] and Carrillo, Colom, Suñol and Saurina [2].

Sample	Wavelength (cm^{-1})	Assignment
Polyester	2961	CH_2 asymmetric stretching
	2855	CH_2 asymmetric stretching

	1712	C=O stretching
	1504	C=C aromatic stretching
	1240	C-O esteric stretching
	1092	
	1016	aromatic ring in-plane CH bend
	721	aromatic in-phase CH wag
Viscose/Lyocell	3309-3310	O-H stretching intramolecular hydrogen bonds
	2887-2891	CH_2 asymmetric stretching
	1638	OH of water absorbed from cellulose
	1417-1418	CH_2 symmetric bending
	1366-1367	CH bending
	1333-1335	–OH in plane bending
	1313	CH_2 wagging
	1199-1200	–OH in plane bending
	1155	C–O–C asymmetric stretching
	1017	C-O stretching
	992-994	
	893-894	γ (COC) in plane, symmetric stretching

S3. Characterisation of textile fibres by Py-GCxGC-TOF MS

The resulting pyrolysis-gas chromatography x gas chromatography- time of flight mass spectrometry (Py-GCxGC-TOF MS) chromatograms and main pyrolysis products detected are shown for polyester (Figure S5a, Table S2), viscose (Figure S5b, **Error! Reference source not found.**Table S3) and lyocell (Figure S5c, Table S4).

Py-GCxGC-TOF MS data for the three materials present pyrolysis products consistent with the chemical composition of the three materials. Polyester samples present mostly aromatic species, such as terephthalates, and PET pyrolysis markers like vinyl benzoate [3]. The pyrolysis products of viscose and lyocell samples include mainly aldehydes, ketones, furans and anhydrosugars (like levoglucosan and 1,4:3,6-dianhydro-α-D-glucopyranose) with their derivatives, typical compounds of cellulose-based materials [4]. Py-GCxGC-TOF MS analysis did not detect the presence of additives, dyes or finishing products in any of the materials studied.

a)

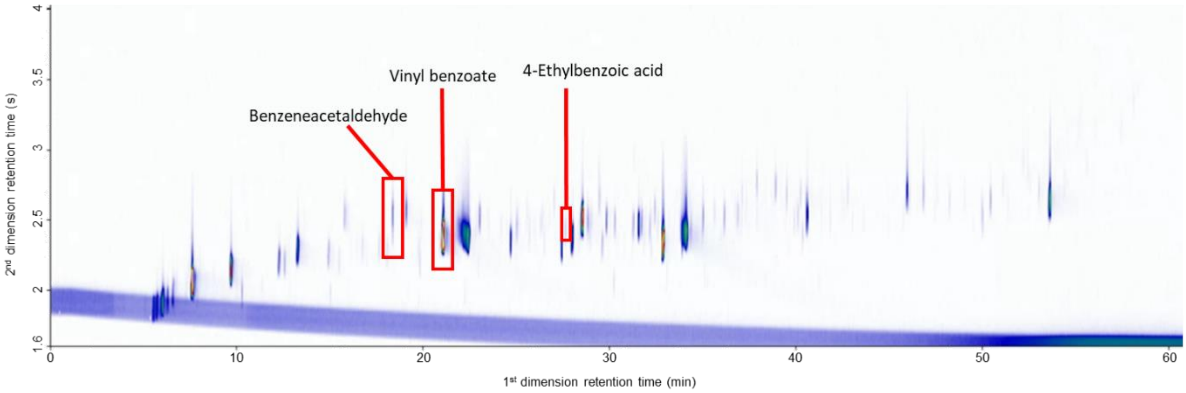

Benzeneacetaldehyde
Vinyl benzoate
4-Ethylbenzoic acid
2nd dimension retention time (s)
1st dimension retention time (min)

b)

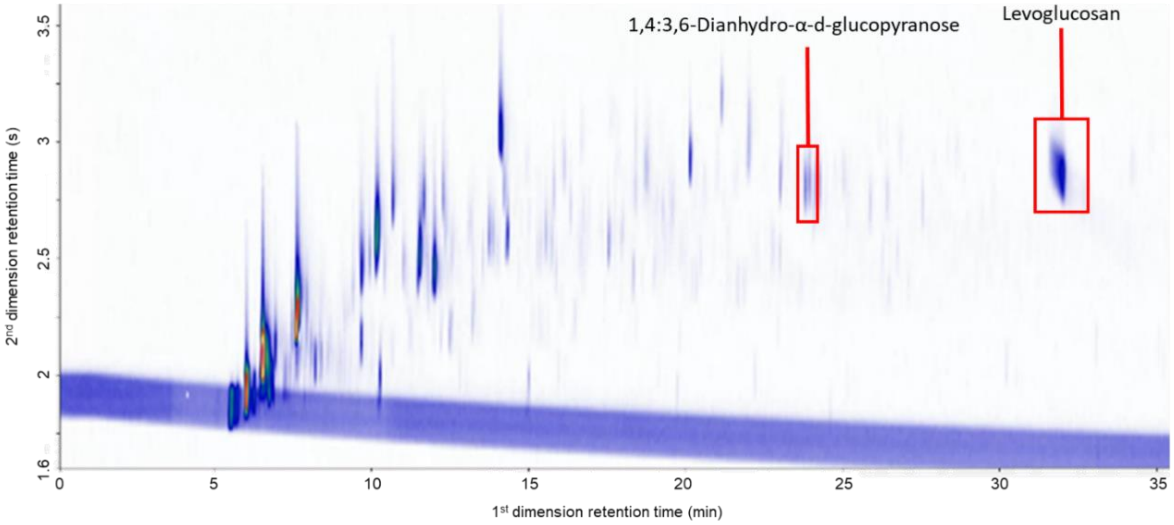

1,4:3,6-Dianhydro-α-d-glucopyranose
Levoglucosan
2nd dimension retention time (s)
1st dimension retention time (min)

c)

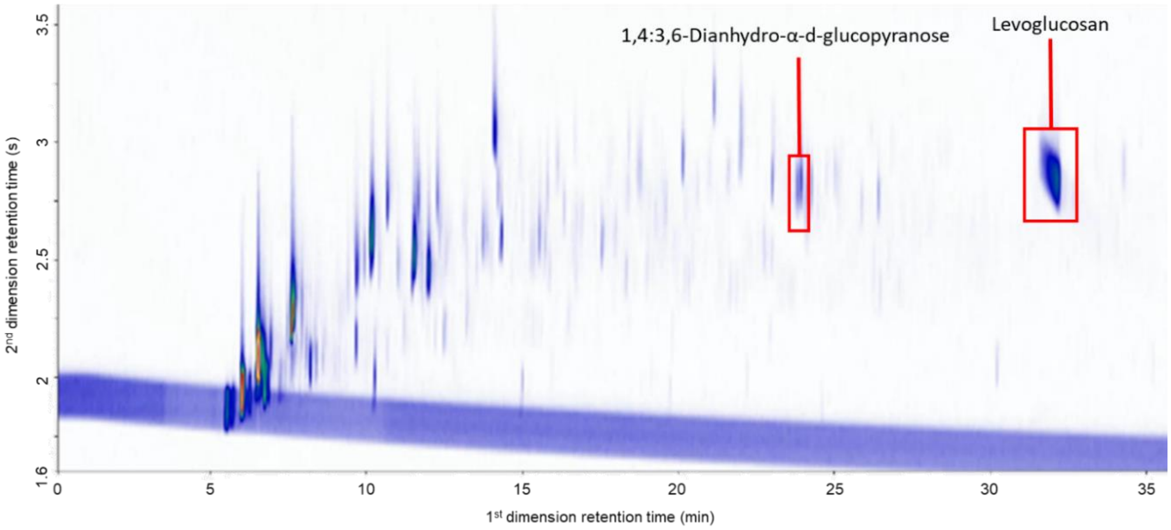

Figure S5. Py-GCxGC-TOF MS chromatogram at 600 °C of a) polyester, b) viscose, and c) lyocell samples with some polymer-specific pyrolysis markers highlighted. Identification tables are reported Table S2 -Table S4.

Table S2. Main pyrolysis products detected in the pyrograms of polyester samples. The pyrogram is shown in Figure S5a.

${}^1t_R$ *(min)*	${}^2t_R$ *(s)*	*Compound*	*Main ions (m/z)*
5.5	1.9	Carbon dioxide	**44**
7.6	2.1	Benzene	39, 51, 63, **78**
9.7	2.1	Toluene	65, **91**, 92
12.2	2.2	Ethylbenzene	51, 65, 77, **91**, 106
12.6	2.2	p-Xylene	39, 51, 77, **91**, 106
13.2	2.3	Styrene	51, 63, 78, **104**
14.9	2.3	Benzene, 2-propenyl-	51, 65, 91, **117**
15.5	2.2	Benzene, 1-ethyl-4-methyl-	77, 91, **105**, 120
15.8	2.5	Benzaldehyde	51, 77, **105**, 106
16.7	2.3	Benzene, 1-ethenyl-3-methyl-	58, 91, 103, **118**
17.6	2.3	Benzene, 2-propenyl-	51, 58, 77, 91, **117**
18.3	2.5	Benzeneacetaldehyde	65, **91**, 120
19.1	2.6	Acetophenone	51, 77, **105**, 120
20.9	2.3	Benzene, 1,3-diethenyl-	51, 77, 102, 115, **130**
21.0	2.4	Vinyl benzoate	51, 77, **105**, 148
22.4	2.4	Benzoic acid	51, 77, **105**, 122
23.0	2.5	Naphthalene	51, 63, 74, 102, **128**
24.7	2.4	Benzoyl chloride, 2-methyl-	39, 65, 91, **119**, 154
25.0	2.4	Benzoic acid, 4-methyl-	65, **91**, 119, 136
27.0	2.6	Ethanone, 2-hydroxy-1-phenyl-	51, 77, **105**, 136
27.4	2.3	Benzoic acid, 4-ethyl-, 4-cyanophenyl ester	79, 105, **133**
27.6	2.4	4-Ethylbenzoic acid	77, 91, **105**, 107, 135, 150
28.0	2.4	1-Penten-3-one, 4-methyl-1-phenyl-	51, 77, 103, **131**, 174
28.4	2.5	4-Vinylbenzoic acid	51, 77, 103, 131, **148**
28.5	2.5	Biphenyl	76, 153, **154**
28.8	2.5	3-Bromophthalide	51, 77, 105, **133**

29.4	2.6	1,2-Ethanediol, monobenzoate	51, 77, **105**, 123
29.6	2.3	2-Propenal, 2-methyl-3-phenyl-	51, 63, 91, 115, **145**
29.8	2.5	Diphenylmethane	91, 153, 165, **168**
30.3	2.5	Diethylene glycol dibenzoate	77, 105, **149**
31.5	2.5	1,1'-Biphenyl, 4-methyl-	152, 165, **168**
31.7	2.5	Ethanone, 1,1'-(1,3-phenylene)bis-	43, 91, 119, **147**, 162
32.2	2.5	Bibenzyl	65, **91**, 182
32.4	2.6	Benzoyl chloride, 4-propyl-	91, 119, **147**
32.9	2.4	Ethylphenylhydantoin	77, 104, **175**
34.1	2.4	4-Acetylbenzoic acid	65, 121, **149**
35.0	2.5	1,1'-Biphenyl, 4-ethenyl-	76, 165, **180**
36.8	2.5	Stilbene	76, 89, 152, 165, **180**
37.9	2.8	9H-Fluoren-9-one	76, 152, **180**
38.9	2.7	Phenanthrene	89, 152, **178**
40.2	2.7	1H-Indene, 1-(phenylmethylene)-	76, 89, 101, **203**
40.5	2.5	p-Phenylphenacyl chloride	76, 152, **181**, 230
40.6	2.5	4-Phenylbenzhydrazide	75. 152, **181**, 212
45.9	2.7	1,2-Ethanediol, dibenzoate	51, 77, **105**
46.1	2.7	m-Terphenyl	**230**
46.8	2.7	p-Terphenyl	**230**

Table S3. Main pyrolysis products detected in the pyrograms of viscose samples. The pyrogram is shown in Figure S5b.

$^{1}t_R$ *(min)*	$^{2}t_R$ *(s)*	***Compound***	***Main ions (m/z***
5.5	1.9	Carbon dioxide	**44**
6.0	1.9	Acetone	**43**, 58
6.3	1.9	1,3-Cyclopentadiene	32, 39, 40, 65, **66**
6.5	2.1	Acetaldehyde, hydroxy-	**31**, 32, 42, 60
6.7	1.9	Furan, 2-methyl-	39, 53, **82**
7.5	2.2	2-Butenal	39, 41, **70**
7.6	2.2	2-Propanone, 1-hydroxy-	31, 43, 74
8.1	2.1	2,3-pentanedione	**43**, 5, 71, 100
8.2	2.0	Furan, 2,5-dimethyl-	43, 53, 67, 81, 95, **96**
8.6	2.1	2-Vynilfuran	39, 65, **94**
9.7	2.5	Acetic acid, (acetyloxy)-	**43**, 73
9.7	2.2	Toluene	51, 65, **91**, 92
9.9	2.5	2(5H)-Furanone	39, **55**, 84
10.4	2.5	Cyclopentanone	41, **55**, 84
11.6	2.5	Furfural	39, 67, 95, **96**
11.7	2.8	2-Cyclopenten-1-one	39, 54, **82**
12.0	2.5	2-Furanmethanol	41, 53, 69, 81, **98**
12.3	2.8	2-butanone	**43**, 57, 72
12.3	2.7	2-Propanone, 1-(acetyloxy)-	**43**, 86, 116
12.5	2.2	p-Xylene	51, 65, 77, **91**, 106
13.1	2.7	4-Cyclopentene-1,3-dione	42, 54, 68, **96**
13.2	2.3	Styrene	51, 78, **104**

13.8	2.6	2-Cyclopenten-1-one, 2-methyl-	39, 53, 67, **96**
13.9	2.6	Ethanone, 1-(2-furanyl)-	43, 67, **95**, 110
14.1	3.0	Cyclopentanone	42, 55, 84
14.3	2.6	2-Cyclopenten-1-one, 2-hydroxy-	42, 55, 69, **98**
15.6	2.6	2-Furancarboxaldehyde, 5-methyl-	53, 81, 109, **110**
15.7	2.6	Benzaldehyde	39, 51, 77, **105**
15.8	2.8	2-Cyclopenten-1-one, 3-methyl-	41, 53, 67, 81, **96**
16.1	2.9	2(5H)-Furanone, 3-methyl-	41, 69, 98
17.6	2.6	2-Cyclopenten-1-one, 2-hydroxy-3-methyl-	41, 55, 69, 83, **112**
18.0	2.6	2,3-dimethyl-2-cyclopenten-1-one	54, 67, 95, **110**
18.3	2.4	1-propynyl-benzene	51, 63, 89, **115**, 116
23.0	2.5	Naphthalene	51, 64, 102, 128
23.0	2.9	1-Pentanol, 2,3-dimethyl-	**43**, 57, 69, 85
23.8	2.8	1,4:3,6-Dianhydro-α-d-glucopyranose	41, 57, **69**, 85, 97, 109, 124, 144
23.9	2.8	5-Hydroxymethylfurfural	41, 53, 69, 81, **97**, 109, 126
24.2	2.8	3,4-Anhydro-d-galactosan	43, 57, **71**, 81, 97, 144
25.9	2.7	1H-Inden-1-one, 2,3-dihydro-	51, 63, 78, 104, **132**
32.0	2.9	Levoglucosan	43, 57, **60**, 70, 73, 98

Table S4. Main pyrolysis products detected in the pyrograms of lyocell samples. The pyrogram is shown in Figure S5c.

$^{1}t_{R}$ (min)	***$^{2}t_{R}$ (s)***	***Compound***	***Main ions (m/z)***
5.5	1.8	Carbon dioxide	**44**
6.0	1.9	Acetone	**43**, 58
6.2	1.9	1,3-Cyclopentadiene	39, 40, 65, **66**
6.5	1.9	Furan, 2,5-dihydro-	39, 41, **70**
6.6	2.1	Acetaldehyde, hydroxy-	**31**, 32, 42, 60
6.7	1.9	Furan, 2-methyl-	39, 53, **82**
7.5	2.2	Furan, 2,3-dihydro-	39, 41, 69, **70**
7.6	2.1	Benzene	39, 52, **78**
7.7	2.3	2-Propanone, 1-hydroxy-	31, **43**, 74
7.7	2.1	3-Penten-2-one	39, **41**, 43, 69, 84
8.2	2.0	Furan, 2,5-dimethyl-	43, 53, 67, 81, 95, **96**
8.6	2.1	2-Vinylfuran	39, 65, **94**
9.7	2.1	Toluene	51, 65, **91**, 92
9.7	2.5	Acetic acid, (acetyloxy)-	**43**, 73
9.9	2.5	2(5H)-Furanone	39, **55**, 84
10.4	2.5	Cyclopentanone	41, **55**, 84
11.2	2.2	Phenol, 3-methyl-	53, 77, 79, 107, **108**
11.5	2.5	Furfural	39, 67, **95**, 96
11.7	2.7	2-Cyclopenten-1-one	39, 54, **82**
12.0	2.5	2-Furanmethanol	41, 53, 69, 81, **98**
12.3	2.8	2-butanone	**43**, 57, 72
12.4	2.7	2-propanone, 1-(acetyloxy)-	**43**, 86, 116

12.5	2.2	p-Xylene	51, 65, 77, **91**, 106
13.2	2.3	Styrene	51, 78, **104**
13.8	2.6	2-methyl-2-cyclopenten-1-one	39, 53, 67, **96**
13.9	2.6	Ethanone, 1-(2-furanyl)-	43, 67, **95**, 110
14.1	3.0	Cyclopentanone	42, 55, 84
14.3	2.6	1,2-Cyclopentanedione	2, 55, 69, **98**
15.6	2.6	2-Furancarboxaldehyde, 5-methyl-	39, 53, 81, 109, **110**
15.7	2.6	Benzaldehyde	39, 51, 77, **105**
15.9	2.5	Phenol	50, 65, **94**
17.5	2.6	2-hydroxy-3-methyl-2-cyclopenten-1-one	41, 55, 69, 83, **112**
18.0	2.6	2,3-dimethyl-2-cyclopenten-1-one	54, 67, 95, **110**
18.3	2.4	1-propynyl-benzene	51, 63, 89, **115**, 116
20.3	2.4	Benzofuran, 2-methyl-	51, 63, 77, 103, **131**, 132
23.0	2.5	Naphthalene	51, 64, 102, **128**
23.0	2.8	1-Pentanol, 2,3-dimethyl-	**43**, 57, 69, 85
23.8	2.8	1,4:3,6-dianhydro-α-D-glucopyranose	41, 57, **69**, 85, 97, 109, 124, 144
24.0	2.8	5-Hydroxymethylfurfural	41, 53, 69, **97**, 109, 124
24.3	2.8	2,3-Anhydro-d-mannosan	43, 55, **71**, 97
25.9	2.7	1H-Inden-1-one, 2,3-dihydro-	51, 63, 78, 104, **132**
26.8	2.5	Naphthalene, 1-methyl-	63, 70, 115, 141, **142**
32.1	2.8	levoglucosan	43, 57, **60**, 70, 73, 98
34.3	2.9	1,6-Anhydro-α-d-galactofuranose	43, 57, 69, **73**, 85, 115

S4. Powder X-ray diffraction

In order to determine the crystallinity of materials, powder x-ray diffraction (PXRD) measurements were performed on a Stoe Stadi P X-ray diffractometer in transmission mode, using Cu-Kα1 radiation at 40 kV and 40 mA. The data collection covered a range of 2.000 to 75.365^{o} in 2 theta and was recorded with multi-Mythen detectors.

The intensity and surface of the peak at 20-21° in 2 theta of Lyocell was higher than that for viscose implying lyocell is more crystalline than viscose[5] (Figure S6).

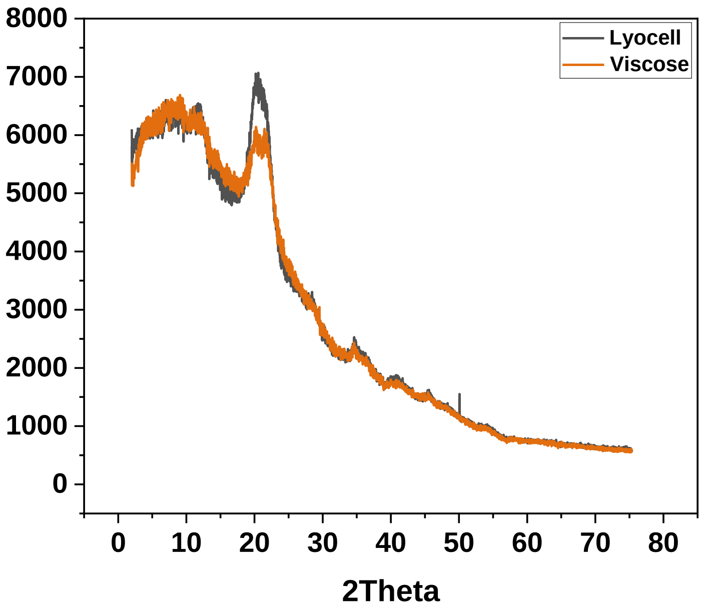

Figure S6: PXRD patterns of Lyocell and Viscose.

S5. Fibre enumeration

The concentration of fibres are expressed as numerical concentrations (fibres.mL) as derived via a Sedgewick Rafter counting chamber [6], and as mass concentrations acquired by weighing the fibres. Both values are summarised to aid comparison with environmental monitoring studies and provide the pertinent information for regulatory assessment.

The mass and numerical concentration of biobased fibres (lyocell and viscose) align tightly, however, polyester fibres had a higher mass when normalised by number (Table S5 and Figure S7).

Table S5. Comparison of the numerical and mass concentrations of fibres for each material.

**Polymer**	**Number of fibres (fibres.mL^{-1})**	**Mass of fibres (mg.mL^{-1})**
Viscose	10000	0.71667
	3200	0.23333
	1000	0.07167
	320	0.02333
	100	0.00717
Lyocell	10000	0.72500
	3200	0.20000
	1000	0.07250
	320	0.02000
	100	0.00725
Polyester	10000	3.96662
	3200	1.47500
	1000	0.26667

	320	0.14750
	100	0.02667

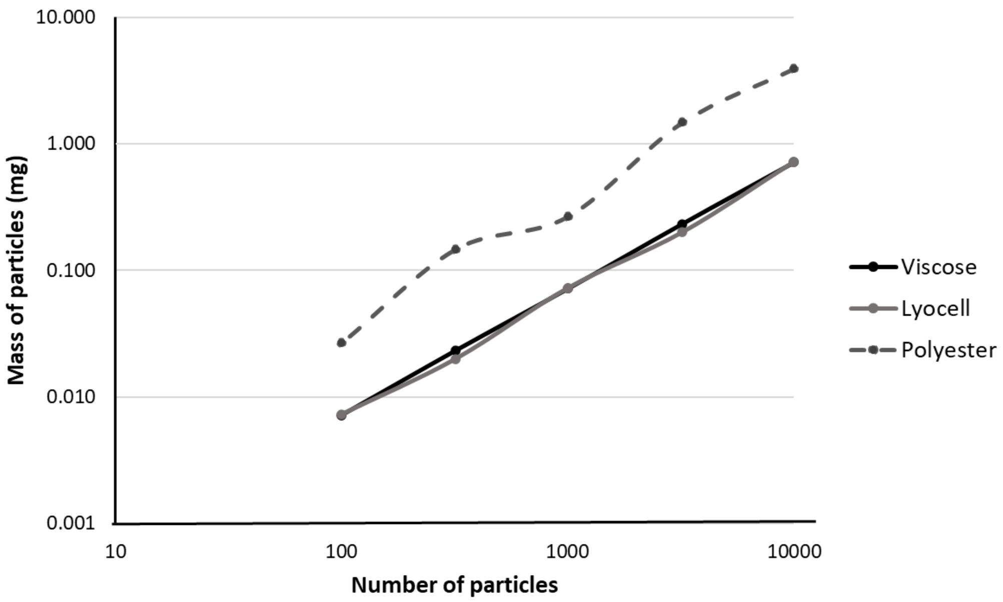

Figure S7. The mass concentration (mg.mL^{-1}) plotted against the measured number concentration (fibres.mL^{-1}) of microplastic fibre suspensions.

S6. Lethal toxicity of 3,4-Dichloroaniline

3,4-Dichloroaniline (3,4-DCA) was used as a reference chemical to ensure that earthworms were responsive to the test system. A concentration series of 3,4-DCA was prepared in ultrapure water (0, 6.36, 20.36, 63.62, 203.58, 636.20 µg.mL^{-1}) and earthworms were exposed (*n* = 5) for 48 hours according to the filter paper contact test method [7]. Dose response curves were constructed (Figure S8) and the lethal concentrations was computed (Table S6), using Sigmaplot V14.5 software.

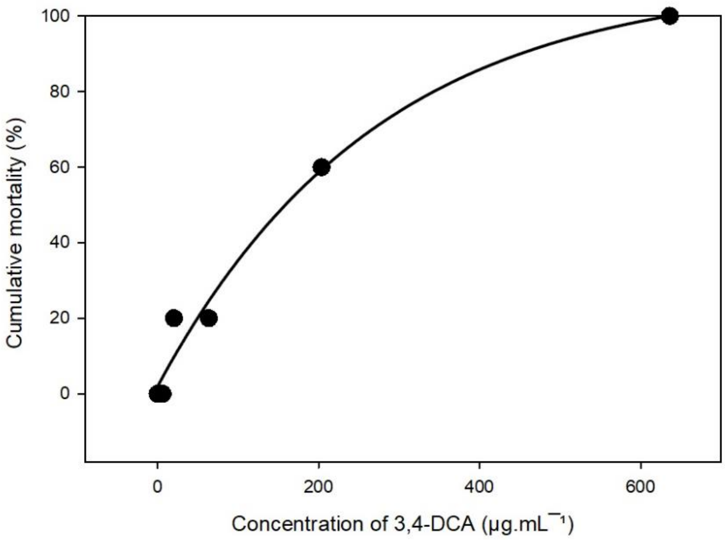

Figure S8. Dose-response plot of the percentage cumulative mortality of *Eisenia fetida* following 48-hour exposure to a concentration series of 3,4-DCA (µg.mL^{-1})

Table S6. The lethal concentration (± standard error) of 3,4-DCA inducing 10 %, 20 % and 50 % mortality in *E. fedita* following 48-hour exposure.

	Concentration of 3,4-DCA (µg.mL^{-1})
LC_{10}	5.811
LC_{20}	9.553
LC_{50}	20.023

S7. Supplementary method for determination of total glutathione concentrations

Total Glutathione (GSH), a component of the antioxidant defence system, was quantified in triplicate for each sample (n = 8 homogenates per treatment) according to Owens and Belcher [8]. A sub-sample of 20 µl of diluted homogenate, blank or standard was pipetted into a reaction mixture (290µl), containing HEPES (50mM) and ethylenediaminetetraacetic acid or EDTA (5mM) buffered to pH 7.5 with sodium hydroxide (2M), 5,5-dithiobis-(2-nitrobenzoicacid) or DTNB (1mM), dihydronicotinamide-adenine dinucleotide phosphate or NADPH (1mM) and glutathione reductase (0.1 U ml^{-1}, from *Saccharomyces cerevisiae,* Sigma G3664), mixed using the shake function of the Spectramax Plus 384 plate reader (Molecular Devices, UK). The change in absorbance was measured at 4 second intervals over 10 minutes at 412 nm. The total glutathione concentration in each homogenate was determined using the standard calibration curve (a dilution series of 2mM reduced glutathione standard).

Total protein was determined using the Pierce BCA kit (#RE232674, Thermo Scientific, UK) using 25 µl of homogenate (in triplicate) and 200 µl of working reagent according to the manufacturer's instructions against bovine serum albumin standards (ranging from 0 -2 g l^{-1} with linear fit, $r^2 > 0.9$).

S8. Results: Oxidative stress

The concentration of total glutathione, a component of the antioxidant defence system, did not statistically differ between treatment groups or between exposure time (Figure S9).

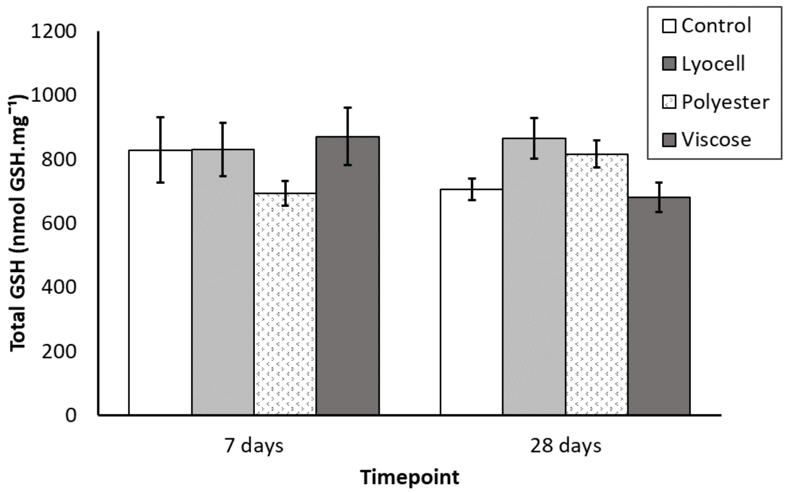

Figure S9. The total glutathione (GSH) concentration, expressed as nmol per mg protein, in *E. fetida* following 7- and 28-days exposure to soils containing different microfibres (100 mg.kg^{-1}). Data are mean (n = 8) ± SEM.

S9. References

(1) Noda, I.; Dowrey, A. E.; Haynes, J. L.; Marcott, C. Group Frequency Assignments for Major Infrared Bands Observed in Common Synthetic Polymers. In *Physical Properties of Polymers Handbook*, Mark, J. E. Ed.; Springer New York, 2007; pp 395-406.
(2) Carrillo, F.; Colom, X.; Suñol, J. J.; Saurina, J. Structural FTIR analysis and thermal characterisation of lyocell and viscose-type fibres. *European Polymer Journal* **2004**, *40* (9), 2229-2234. DOI: https://doi.org/10.1016/j.eurpolymj.2004.05.003.
(3) Wu, T.; Hu, H. L.; Du, Y. P.; Jiang, D.; Yu, B. H. Discrimination of Thermoplastic Polyesters by MALDI-TOF MS and Py-GC/MS. *International Journal of Polymer Analysis and Characterization* **2014**, *19* (5), 441-452. DOI: 10.1080/1023666X.2014.920126.
(4) Lu, Q.; Yang, X.-c.; Dong, C.-q.; Zhang, Z.-f.; Zhang, X.-m.; Zhu, X.-f. Influence of pyrolysis temperature and time on the cellulose fast pyrolysis products: Analytical Py-GC/MS study. *Journal of Analytical and Applied Pyrolysis* **2011**, *92* (2), 430-438. DOI: https://doi.org/10.1016/j.jaap.2011.08.006.
(5) Siroka, B.; Noisternig, M.; Griesser, U. J.; Bechtold, T. Characterization of cellulosic fibers and fabrics by sorption/desorption. *Carbohydr Res* **2008**, *343* (12), 2194-2199. DOI: 10.1016/j.carres.2008.01.037.
(6) Intergovernmental Oceanographic Commission of UNESCO. *Microscopic and molecular methods for quantitative phytoplankton analysis*; UNESCO, 2010.
(7) OECD. *OECD Guideline for the testing of chemicals, Technical guidance 207, Earthworm acute toxicity test*; 1984.
(8) Owens, C.; Belcher, R. A colorimetric micro-method for the determination of glutathione. *Biochemical Journal* **1965**, *94* (3), 705-711. DOI: 10.1042/bj0940705 (acccessed 2/17/2023).
